# Effect of intermittent Pringle maneuver on perioperative outcomes and long-term survival following liver resection in patients with hepatocellular carcinoma: a meta-analysis and systemic review

**DOI:** 10.1186/s12957-023-03244-x

**Published:** 2023-11-21

**Authors:** Lingbo Hu, Aidong Wang, Yingli Qiao, Xiandan Huang

**Affiliations:** 1 Department of Hepatopancreatobiliary Surgery, Taizhou Hospital of Zhejiang Province Affiliated to Wenzhou Medical University, Zhejiang, China; 2 Department of Hepatopancreatobiliary Surgery, Taizhou Enze Medical Center (Group), Enze Hospital, Zhejiang, China

**Keywords:** Pringle maneuver, Liver resection, Hepatocellular carcinoma, Long-term survival, Meta-analysis

## Abstract

**Background:**

Intermittent Pringle maneuver (IPM) is commonly used to control bleeding during liver resection. IPM can cause ischemia–reperfusion injury, which may affect the prognosis of patients with hepatocellular carcinoma (HCC). The present meta-analysis was conducted to evaluate the effect of IPM use on perioperative outcomes and long-term survival in patients with HCC.

**Methods:**

A systemic literature search was performed in the PubMed, Embase, Web of Science, and Cochrane Library databases to identify randomized controlled trials and retrospective studies that compared the effect of IPM with no Pringle maneuver during liver resection in patients with HCC. Hazard ratio (HR), risk ratio, standardized mean difference, and their 95% confidence interval (CI) values were calculated based on the type of variables.

**Results:**

This meta-analysis included nine studies comprising one RCT and eight retrospective studies and involved a total of 3268 patients. Perioperative outcomes, including operation time, complications, and length of hospital stay, except for blood loss, were comparable between the two groups. After removing the studies that led to heterogeneity, the results showed that IPM was effective in reducing blood loss. Five studies reported overall survival (OS) and disease-free survival (DFS) data and eight studies reported perioperative outcomes. No significant difference in OS and DFS was observed between the two groups (OS: HR, 1.01; 95% CI, 0.85–1.20; *p* = 0.95; DFS: HR, 1.01; 95% CI, 0.88–1.17; *p* = 0.86).

**Conclusion:**

IPM is a useful technique to control blood loss during liver resection and does not affect the long-term survival of patients with HCC.

**Supplementary Information:**

The online version contains supplementary material available at 10.1186/s12957-023-03244-x.

## Introduction

Liver resection is the most widely used curative treatment approach for hepatocellular carcinoma (HCC) as recommended by several guidelines [1–3]. The control of bleeding during liver resection is an important aspect to ensure surgical safety and reduce complications [4]. Current evidence shows that massive blood loss during liver resection and blood transfusion are the risk factors for poor overall survival (OS) and disease-free survival (DFS) of patients undergoing liver resection [5, 6]. Intermittent Pringle maneuver (IPM) is the most common vascular occlusion method used worldwide to control bleeding [7]. The clamping pattern of IPM included 15 min of ischemia followed by 5 min of reperfusion. However, blood flow occlusion inevitably causes ischemia–reperfusion injury in the liver, resulting in liver function damage [8] and contributing to HCC recurrence [9, 10]. Therefore, a part of hepatectomies was done without the Pringle maneuver.

The utility of IPM in reducing bleeding during liver resection was evaluated [11]. The effect of IPM on the long-term survival of patients with liver malignancies was assessed [12]. However, these studies did not focus on the effect of IPM on short- and long-term outcomes following hepatectomy specifically for HCC patients. It is known that patients with HCC usually have hepatitis or cirrhosis or other chronic liver diseases as coexisting illnesses. Consequently, the effects of IPM on these patients may differ from those on patients with normal liver [13]. Hence, we conducted a meta-analysis to evaluate the effect of IPM on perioperative outcomes and long-term survival of patients with HCC who underwent liver resection.

## Methods

This meta-analysis was performed using the Preferred Reporting Items for Systematic Reviews and Meta-Analyses (PRISMA). This systematic review is registered in the PROSPERO database (registration no. CRD42023411488).

### Article search strategy

A systematic search was independently performed by two researchers to identify the relevant studies published on this topic in the PubMed, Embase, Web of Science, and Cochrane Library databases from conception to March 26, 2023. The following MeSH terms and keywords were used for search in PubMed: liver resection, hepatectomy, hepatic resection, hepatocellular carcinoma, Pringle maneuver, and blood occlusion. The details of search strategies are provided in Supplementary Material 1. Manual retrieval was performed to identify eligible studies from the included studies, meta-analyses, and reviews.

### Selection criteria

The inclusion criteria were as follows: (1) studies involving patients with HCC; (2) studies on comparison between the use of IPM and no use of PM (non-Pringle maneuver [NPM]) during liver resection, both randomized controlled trials (RCTs) or retrospective studies published in English; and (3) studies reporting perioperative outcomes (including blood loss, blood transfusion, and complications) and/or long-term outcomes (including OS and DFS).

The exclusion criteria were as follows: (1) studies that included patients with liver cancers or benign tumors other than HCC; (2) studies that compared other blood occlusion methods, including continuous PM, selective hepatic flow occlusion, or hemihepatic blood flow occlusion; and (3) studies with duplicate data.

### Quality assessment and data extraction

Quality assessment and data extraction were conducted by two independent researchers. The Cochrane risk assessment tool was used to assess the quality of RCTs. The Newcastle–Ottawa Scale with a score of up to 9 points was used to assess the quality of retrospective studies; the scores were assigned as follows: 5 or less, low quality; 6–7, moderate quality; and 8 or more, high quality.

Predesigned and standardized forms were used to extract relevant details from the included studies (first author, country, year of publication, patient information, and tumor characteristics). The survival outcomes, including OS, DFS, and recurrence rate, and perioperative outcomes, including operation time, blood loss, blood transfusion, and complications were extracted. Survival data were collected directly from the original reports or indirectly from estimation with the Kaplan–Meier curve using the Engauge Digitizer software (version 4.1). Any disagreements between the two independent researchers were resolved by a third researcher.

### Statistical analysis

The inverse variance method was used to determine the hazard ratio (HR) and 95% confidence interval (CI) values. The Mantel–Haenszel method was used to determine the risk ratio (RR) and 95% CI values. The Hedges method was used to calculate the standardized mean difference (SMD) and 95% CI values. Heterogeneity was assessed using the *χ*^2^ method (*I*^2^ values of 25% and 50% indicated low heterogeneity and moderate heterogeneity, respectively) [14]. The test model was selected based on the heterogeneity level, and the random-effects model was used for *I*^2^ > 50%. Sensitivity analysis was performed to assess the robustness of the conclusion. The publication bias was determined using funnel plots. The trim and fill method was used if an apparent publication bias was noted. A *p* value of < 0.05 indicated statistical significance. All statistical analyses were conducted in R program (version 4.2.3).

## Results

### Study search and selection details

**Fig. 1:**
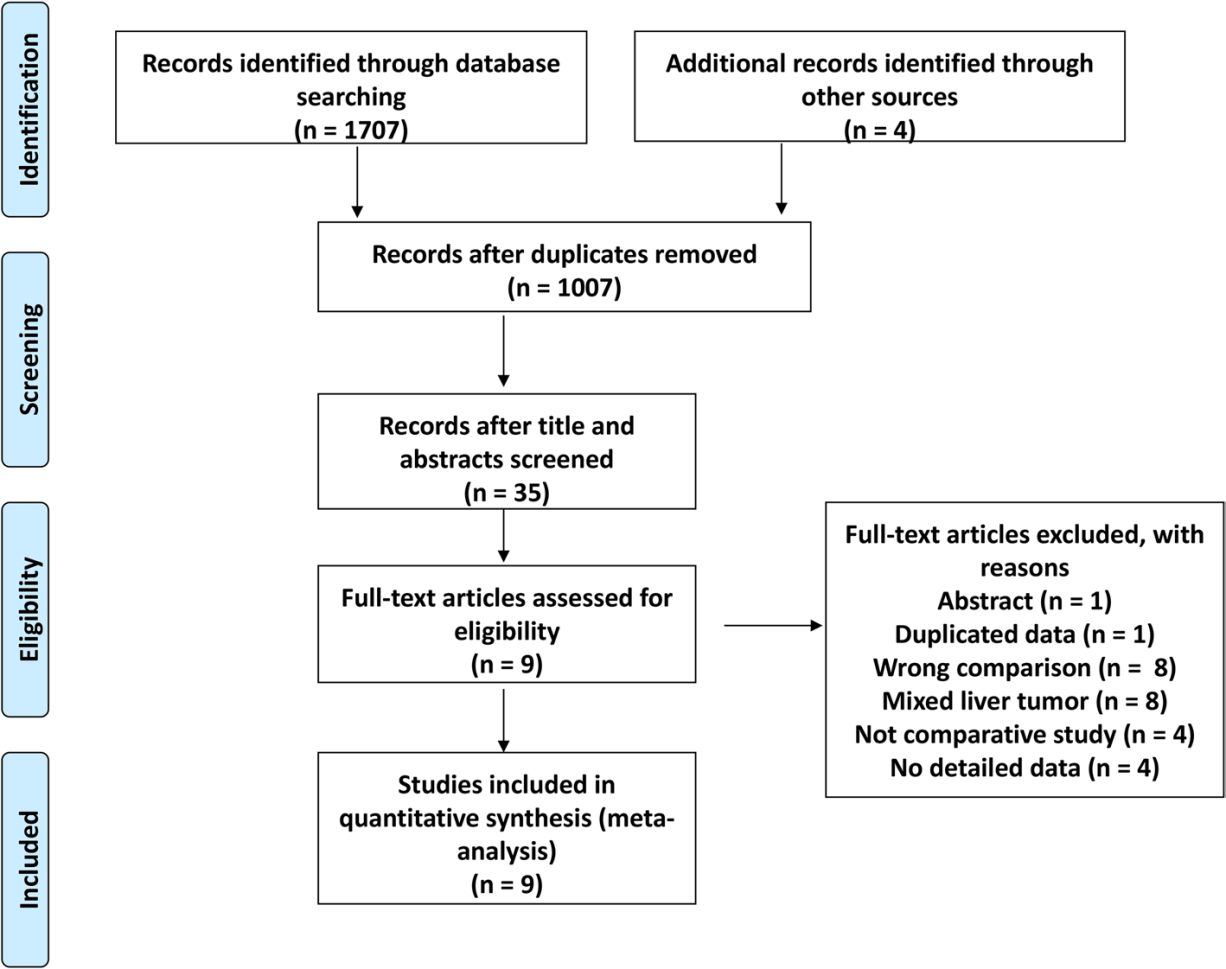
Flow chart of study selection

Our database search yielded 1007 records after duplicate studies were removed. The titles and abstracts of these records were screened for their relevance, and 35 articles were retained for further evaluation. Of these 35 articles, 26 articles were further excluded because of incorrect comparison (*n* = 8), mixed liver tumor (*n* = 8), noncomparative study (*n* = 4), lack of detailed data (*n* = 4), duplicate data (*n* = 1), or study presented as an abstract (*n* = 1). Thus, 9 studies were selected for the meta-analysis [15–23] (Fig. 1).

### Study characteristics

The included 9 studies comprising one RCT and 8 retrospective studies and involved a total of 3268 patients, of which 1703 patients underwent liver resection with IPM (Table 1). Five studies reported OS and DFS data and eight studies reported perioperative outcomes. The mean or median occlusion time varied among the studies and ranged from 19.5 to 50 min.

**Table 1:** Characteristics of the included studies

Study	Design	NOS score	Group	Occlusion time (min)	Type of resection Minor/Major	Sample size	Age	Gender M/F	Child–Pugh A/B	Cirrhosis Y/N	HBV/HCV	Tumor size	Tumor number S/M	AFP (ng/ml)	Differentiation W/Mo/P	Survival (months)
Xia [23]	R	8	IPM	50 (30–98)	131/93	224	48 (21–78)	173/51	141/83	169/55	209/NR	6.4 (2.8–20.2)	145/79	143(≥ 400)	NR	mDFS for all patients 26.1
2013			NPM		85/77	162	57 (18–79)	119/43	101/61	128/34	149/NR	5.9 (2.9–21.3)	122/40	93(≥ 400)	NR	
Huang JW [22]	R	7	IPM	NR	374/338	712	56.1 ± 16.55	505/207	NR	518/194	543/59	8.6 ± 7.8	513/199	8176.3 ± 3211.5	NR	NR
2014			NPM		329/289	618	54.2 ± 22.1	473/145	NR	322/296	469/52	7.7 ± 5.1	433/185	6421.2 ± 5641.9	NR	NR
Huang ZP [21]	R	9	IPM	41.9 ± 12.3	0/158	158	48.5 ± 12.0	129/29	NR	NR	134/NR	NR	NR	NR	NR	NR
2014			NPM		0/200	200	50.4 ± 12.6	175/25	NR	NR	155/NR	NR	NR	NR	NR	NR
Hao [20]	R	8	IPM	31.5 ± 12.5	28/50	78	57.1	60/18	48/30	NR	63/NR	32 (≤ 5 cm) 46 (> 5 cm)	47/31	NR	51/27 (W + Mo/P)	OS 19.0 ± 4.2 DFS 14.2 ± 4.6
2016			NPM		25/35	60	53.6	48/12	40/20	NR	50/NR	26 (≤ 5 cm) 34 (> 5 cm)	39/21	NR	37/23	mOS 20.0 ± 3.8 mDFS 18.0 ± 4.8
Hao [19]	R	9	IPM	27.7 ± 7.3	42/71	113	51.7	76/37	74/39	NR	92 (HBV + HCV)	40 (≤ 5 cm) 73 (> 5 cm)	67/46	NR	78/35 (W + Mo/P)	mOS 46.3 mDFS 39.4
2017			NPM		25/27	52	55	37/15	35/17	NR	43 (HBV + HCV)	22 (≤ 5 cm) 30 (> 5 cm)	34/18	NR	32/20	mOS 52.9 mDFS 47.3
Famularo [18]	R	8	IPM	23 (14–30)	153/22	176	65.1 (58.2–72)	145/21	160/15	144/32	NR	119 (< 5 cm) 56 (≥ 5 cm)	133/42	21 (6–132)	NR	mOS 46.4 mDFS 26.7
2018			NPM		228/36	265	67.6 (59.2–73.9)	199/66	248/16	214/51	NR	199 (< 5 cm) 65 (≥ 5 cm)	210/54	18 (6–177)	NR	mOS 56.5 mDFS 24.9
Lee	RCT		IPM	45 (15–87)	51/37	88	58.0 (38.0–84.0)	75/13	88/0	53/25	66/12	3.9 (1.0–12.3)	64/24	28.0 (1.0–191,009.0)	10/73/5	NR
2019			NPM		54/34	88	60.5 (27.0–81.0)	75/13	88/0	50/28	70/3	3.5 (1.0–18.0)	68/20	25.0 (1.0–16,246.0)	5/78/5	NR
Wei [16]	R	9	IPM	50 (30–80)	59/49	108	51.45 ± 12.45	87/21	95/11	40/68	83/NR	NR	NR	55 (> 40)	NR	NR
2019			NPM		44/30	74	49.15 ± 11.10	63/11	65/6	26/48	64/NR	NR	NR	36 (> 40)	NR	NR
Galindo [15]	PSM	9	IPM	19.5 (5–78)	34/12	46	69 (49–85)	36/10	32/2	34/12	4/12	3.1 (0.6–14)	38/8	NR	NR	NR
2022			NPM		36/10	46	68 (19–86)	35/11	31/3	34/12	9/15	3.0 (0.3–9.5)	37/9	NR	NR	NR

*R* retrospective study, *RCT* randomized controlled trial, *PSM* propensity score matched study, *NOS* Newcastle–Ottawa Scale, *IPM* intermittent Pringle maneuver, *NPM* non-Pringle maneuver, *NR* not reported, *M* male,* F* female, *Y* yes, *N* no, *HBV* hepatitis virus B, *HCV* hepatitis virus C, *S* solitary, *M* multiple, *AFP* alpha-fetoprotein, *W* well, *Mo* moderate,* P* poor, *OS* overall survival, *DFS* disease-free survival, *mOS* median overall survival, *mDFS* median overall survival

### Quality assessment results

The risk of bias for studies reporting RCTs was low. The details of the quality assessment of these studies are shown in Supplementary Materials 2 and 3. Among the eight retrospective studies, seven had high quality and one had moderate quality (Table 1).

### OS and DFS analysis

**Fig. 2:**
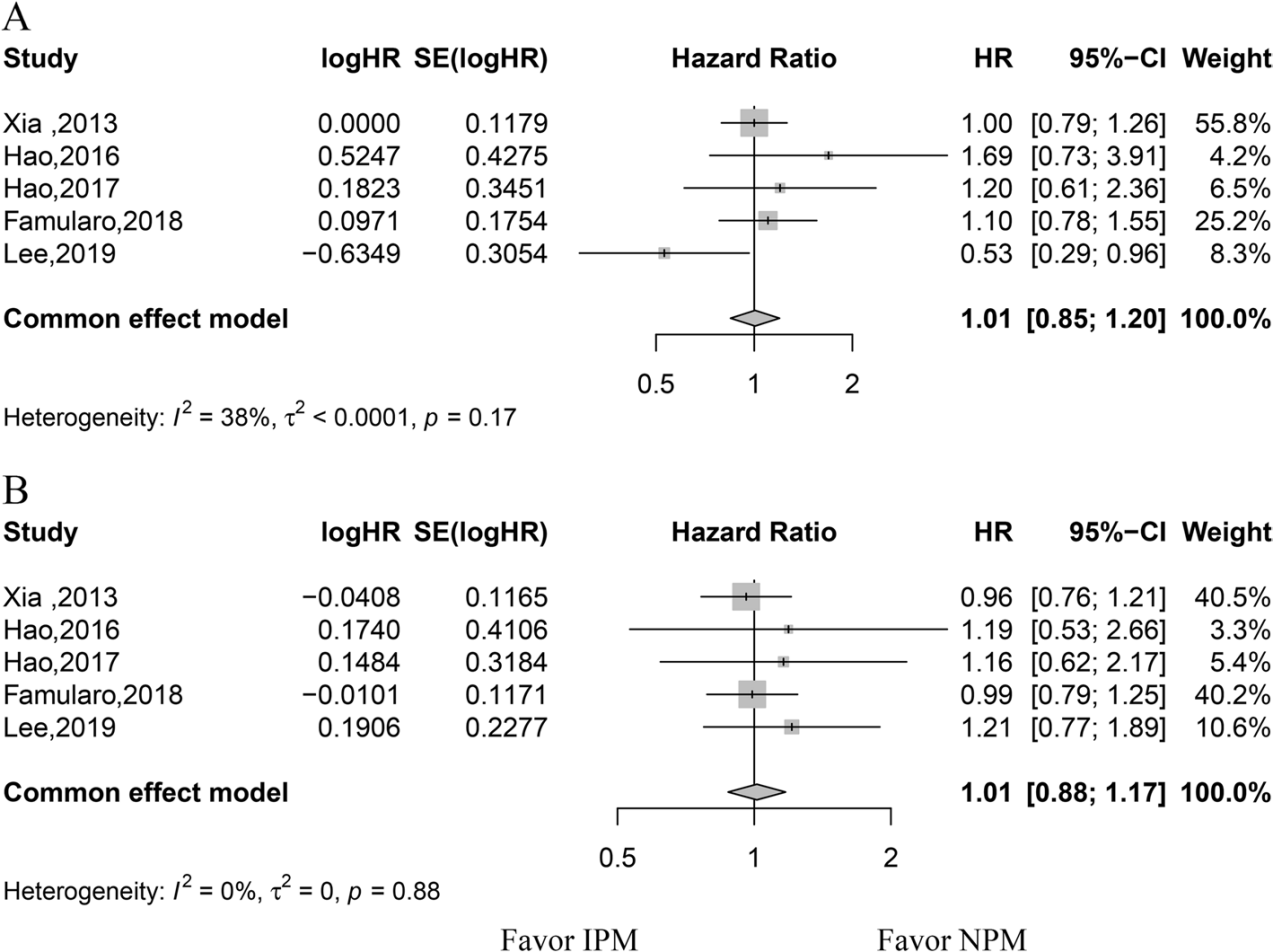
Forest plots for overall survival and disease-free survival. **A** Overall survival forest plot. **B** Disease-free survival forest plot

The HR values of OS and DFS were available for five studies (one RCT and four retrospective studies). Because there was no significant heterogeneity among the studies, the fixed-effects model was used. Because of the larger sample size, the two studies by Xia et al. and Famularo et al. carried the highest impact on the analysis of OS and DFS. The meta-analysis showed no significant difference in the OS and DFS between the two groups (OS: HR, 1.01; 95% CI, 0.85–1.20; *p* = 0.95; DFS: HR, 1.01; 95% CI, 0.88–1.17;* p* = 0.86) (Fig. 2). The results of OS and DFS of subgroup analysis of the retrospective study was consistent with the primary analysis (OS: HR, 1.07; 95% CI, 0.89–1.28; DFS: HR, 0.99; 95% CI, 0.85–1.16) (Supplementary Material 4).

Sensitivity analysis was performed by excluding each study at a time and combining the HR values for the remaining included studies (Supplementary Material 5). Sensitivity analysis showed that excluding studies by Xia et al. and Famularo et al. changed the CIs considerably, but the results of OS and DFS were still stable. Publication bias was considered significant for both OS and DFS because of the presence of asymmetry in the funnel plots (Supplementary Material 6). The trim and fill method was used to evaluate the effect of publication bias on the results of OS and DFS (Supplementary Material 7). The OS and DFS results before and after trimming and filling were similar (OS: HR, 0.98; 95% CI, 0.83–1.16; *p* = 0.84; DFS: HR, 0.99; 95% CI, 0.86–1.13; *p* = 0.83) (Supplementary Material 8).

### Recurrence rate

Four studies reported the tumor recurrence rate. Because of significant heterogeneity among the studies, the random-effects model was used. The meta-analysis showed no significant difference in the tumor recurrence rate between the two groups (RR, 1.22; 95% CI, 0.85–1.74; *p* = 0.28) (Table 2).

**Table 2:** Details of perioperative outcomes and tumor recurrence

Outcomes	Included studies	RR (95% CI)	*p* value	*I*^2^	Model
Blood transfusion	4	1.02 [0.47; 2.20]	0.97	89%	Random
Total complications	5	0.91 [0.70; 1.20]	0.52	71%	Random
Liver failure	6	0.73 [0.35; 1.52]	0.41	0%	Fixed
Pleural effusion	4	1.27 [0.61; 2.64]	0.52	62%	Random
Ascites	3	0.85 [0.46; 1.58]	0.61	57%	Random
Tumor recurrence	4	1.22 [0.85; 1.74]	0.28	69%	Random
		SMD (95% CI)			
Operation time	6	0.23 [− 0.40; 0.86]	0.47	97%	Random
Blood loss	7	0.02 [− 0.30; 0.33]	0.92	95%	Random
Hospital stay	4	0.01 [− 0.28; 0.31]	0.94	78%	Random

*RR* risk ratio, *SMD* standardized mean difference

### Operation time

Operation time was reported in six studies. The random-effects model was used because of significant heterogeneity among the studies. The meta-analysis revealed no significant difference in operation time between both groups (SMD, 0.23; 95% CI, − 0.40 to 0.86; *p* = 0.47) (Table 2).

### Blood loss

Seven studies reported blood loss during liver resection. As there was significant heterogeneity among the studies, the random-effects model was used. The meta-analysis showed no significant difference in the volume of blood loss between both groups (SMD, 0.02; 95% CI, − 0.30 to 0.33; *p* = 0.92) (Table 2). However, sensitivity analysis showed that the study by Fumularo et al. [18] had a great influence on the results including heterogeneity and significance (Supplementary Material 9). The meta-analysis after omitting this study showed that IPM significantly reduced blood loss (SMD, − 0.20; 95% CI, − 0.28 to − 0.12; *p* < 0.01) (Supplementary Material 10). Sensitivity analysis indicated that the result was stable (Supplementary Material 11).

### Blood transfusion

Four studies reported blood transfusion. The random-effects model was used because of significant heterogeneity among the studies. The meta-analysis showed no significant difference in blood transfusion between both groups (RR, 1.02; 95% CI, 0.47–2.20; *p* = 0.97) (Table 2).

### Total complications

Total complications were reported in five studies. Because of significant heterogeneity among the studies, the random-effects model was used. The meta-analysis revealed no significant difference in total complications between both groups (RR, 0.91; 95% CI, 0.70–1.20; *p* = 0.52) (Table 2).

### Liver failure

Liver failure was reported in six studies. The fixed-effects model was used as there was no significant heterogeneity among the studies. The meta-analysis showed no significant difference in liver failure between both groups (RR, 0.73; 95% CI, 0.35–1.52; *p* = 0.41) (Table 2).

### Pleural effusion

Four studies reported pleural effusion. The random-effects model was used because of significant heterogeneity among the studies. The meta-analysis showed no significant difference in pleural effusion between both groups (RR, 1.27; 95% CI, 0.61–1.64; *p* = 0.52) (Table 2).

### Ascites

The occurrence of ascites was reported in three studies. Because of significant heterogeneity among the studies, the random-effects model was used. The meta-analysis showed no significant difference in the occurrence of ascites between both groups (RR, 0.85; 95% CI, 0.46–1.58; *p* = 0.61) (Table 2).

### Hospital stay

The length of hospital stay was reported in four studies. The random-effects model was used as the studies exhibited significant heterogeneity. The meta-analysis showed no significant difference in the length of hospital stay between both groups (SMD, 0.01; 95% CI, − 0.28 to 0.31; *p* = 0.94) (Table 2).

### Subgroup analysis for perioperative outcomes

Subgroup analysis based on the proportion of patients with Child A, the proportion of patients with liver cirrhosis, the proportion of patients receiving major liver resection, and the proportion of patients with multiple tumor resection to explore the sources of heterogeneity for perioperative outcomes.

Subgroup analysis based on the proportion of patients with Child A showed that different proportion of patients with Child A is one source of heterogeneity for operation time, blood loss, blood transfusion, pleural effusion, and hospital stay (Supplementary Material 12). For those studies with a proportion of patients with Child A > 90%, meta-analysis showed that IPM may prolong operation time (SMD, 0.16; 95% CI, 0.01 to 0.37).

Subgroup analysis based on the proportion of patients with liver cirrhosis showed that different proportion of patients with liver cirrhosis is one source of heterogeneity for operation time, blood loss, blood transfusion, total complication, and hospital stay (Supplementary Material 13).

Subgroup analysis based on the proportion of patients who received major liver resection showed that different proportions of patients who received major liver resection are one source of heterogeneity for operation time and blood loss (Supplementary Material 14). For those studies with a proportion of patients who received major liver resection > 60%, a meta-analysis showed that IPM may reduce blood loss (SMD, − 0.23; 95% CI, − 0.41 to − 0.05).

Subgroup analysis based on the proportion of patients who received major liver resection showed that different proportion of patients received major liver resection is one source of heterogeneity for operation time, blood loss, blood transfusion, total complication, pleural effusion, and hospital stay (Supplementary Material 15). For those studies with a proportion of patients with multiple tumors < 25%, meta-analysis showed that IPM may prolong operation time (SMD, 0.16; 95% CI, 0.01 to 0.37). For those studies with the proportion of patients with multiple tumors > 25%, meta-analysis showed that IPM may reduce blood loss (SMD, − 0.17; 95% CI, − 0.34 to − 0.00).

## Discussion

Our present meta-analysis comprehensively analyzed the long-term survival and perioperative effects of IPM on HCC patients undergoing hepatectomy. The results demonstrated that perioperative outcomes, including operation time, blood transfusion, and postoperative complications, except for volume of blood loss, were comparable between the IPM and NPM groups. Additionally, IPM did not improve or worsen the long-term survival of HCC patients who underwent liver resection as compared to NPM.

Regarding perioperative outcomes, we found no significant differences in the volume of blood loss, blood transfusion, operation time, and postoperative complications between both groups; this finding indicated that both IPM and NPM were safe for liver resection. However, the heterogeneity among the included studies was relatively high. Subgroup analysis showed that the proportion of patients with liver cirrhosis, the proportion of patients with Child A, the proportion of patients receiving major liver resections, and the proportion of patients with multiple tumor resections are the source of heterogeneity. These results suggest that the implementation of IPM should take into account the patient’s liver background, the number of tumors, and the volume of the liver removed. For blood loss, we found that the study by Fumularo et al. [18] was one source of heterogeneity. The obvious differences between this study and others were that the relatively short time of PM, the lowest proportion of patients with multiple tumors and patients who received major live resection, and the highest proportion of patients with liver cirrhosis in the study by Fumularo et al. After omitting this study, we found that IPM significantly reduced blood loss compared with NPM. These results may indicate that for these hepatectomies that do require a longer hilar occlusion time, IPM can significantly reduce the volume of blood loss. These results for blood transfusion and postoperative complications in our study were similar to another meta-analysis assessing the value of IPM in reducing bleeding during hepatectomy for both HCC and colorectal liver metastasis [11]. Although several RCTs have studied the effect of IPM on blood loss [24–27], RCTs specifically focused on the effect of IPM on HCC patients are required because these patients often have cirrhosis as a coexisting condition.

IPM could affect the long-term survival of HCC patients in two ways: (1) reduction in intraoperative blood loss and (2) induction of ischemia–reperfusion injury. Increased intraoperative blood loss during HCC resection is an independent prognostic factor for tumor recurrence and death [6]. The subsequent blood transfusion can promote HCC recurrence and reduce long-term patient survival [5]. However, our study found that IPM reduces blood loss for these hepatectomies that do require a longer hilar occlusion time, but did not the need for blood transfusion in HCC patients who underwent liver resection. The amount of blood lost between the two groups did not affect survival, possibly due to the large differences in blood loss values among the included studies. Earlier studies have found that blood loss greater than 1000 ml compared to less than 1000 ml may affect long-term survival in patients with HCC after surgery [6, 28]. However, the average blood loss in most included studies was less than 1000 ml. When blood loss is less than 1000 ml, whether blood loss affects long-term survival is controversial [17, 18]. The choice of cutoff value is important when assessing whether the amount of blood loss affects prognosis. And this value may be different in different studies, so the conclusions will be controversial. On the other hand, different measurements for the amount of blood loss may influence the conclusion. When bleeding occurs and blood transfusion is unavoidable, an allogeneic blood transfusion may also lead to a worse prognosis [29]. Studies on liver transplantation have provided conclusive evidence that ischemia–reperfusion injury affects the long-term survival of patients with liver cancer [30, 31]. Alleviating inflammation caused by ischemia–reperfusion injury can reduce HCC recurrence [9]. However, the time of ischemia occurrence and its characteristics are not completely similar between patients with liver resection and those who have undergone liver transplantation. IPM, continuous PM, selective hepatic flow occlusion, and hemihepatic blood flow occlusion are selected based on tumor characteristics and the preferences of surgeons. The duration of blood flow disruption also varies, which can lead to different survival outcomes for HCC patients [32, 33]. Hence, we compared IPM with NPM in our present study; however, we did not find a significant difference in long-term survival between both groups.

Lin et al. conducted a meta-analysis and concluded that IPM increased the risk of early HCC recurrence but did not affect long-term survival [11]. However, this study had some limitations. Most 1-, 3-, and 5-year OS or DFS rates were extracted from the Kaplan–Meier curve because these data were not reported in the original article; this may have affected the calculated results. Additionally, an inappropriate study was included because the comparison was conducted between NPM and hepatic inflow occlusion (including IPM and selective hemihepatic occlusion) [22]. Wassmer et al. also conducted a meta-analysis on this topic and concluded that PM negatively affected OS and DFS [34]. However, they combined the cases of IPM and continuous PM rather than comparing IPM alone with NPM, which could lead to heterogeneity. Compared with these previous meta-analyses, ours is more homogeneous in terms of inclusion criteria.

The present study has several limitations that should be acknowledged. First, only one RCT was included in our study. Second, relatively high heterogeneity was observed among the studies used for the comparison of perioperative outcomes. Third, regarding long-term survival, publication bias was detected despite low heterogeneity among the studies used for comparison. However, sensitivity analysis showed that the results of OS and DFS were stable; moreover, the trim and fill method revealed that the unpublished studies did not affect the results. Fourth, the number of studies included in the meta-analysis was small.

## Conclusion

Our meta-analysis showed that IPM was a useful technique to control bleeding during liver resection and did not affect the long-term survival of HCC patients.

### Supplementary Information

**Additional file 1:** **Supplementary Material 1.** Search strategy. **Supplementary Material 2.** Risk assessment of RCT. **Supplementary Material 3.** NOS score of non-RCT studies. **Supplementary Material 4.** Subgroup analysis for overall survival and disease-free survival. **Supplementary Material 5.** Sensitivity analyses for overall survival and disease-free survival. **Supplementary Material 6.** Funnel plot for overall survival and disease-free survival. A, overall survival; B, disease-free survival. **Supplementary Material 7.** The effect of publication bias evaluated by using the trim and fill method for overall survival and disease-free survival. **Supplementary Material 8.** Forest plot after trimming and filling for overall survival and disease-free survival. **Supplementary Material 9.** Forest plot of sensitivity analysis for blood loss. **Supplementary Material 10.** Forest plot for blood loss after omitting the study by Fumularo *et al*. **Supplementary Material 11.** Forest plot of sensitivity analysis for blood loss after omitting the study by Fumularo *et al*. **Supplementary Material 12.** Forest plot of subgroup analysis based on the proportion of patients with Child A, the cut value was 90%. A, operation time; B, blood loss; C, blood transfusion; D, total complication; E, pleural effusion; F, hospital stay. **Supplementary Material 13.** Forest plot of subgroup analysis based on the proportion of patients with liver cirrhosis, the cut value was 70% for operation time and blood transfusion, while 60% for the rest. A, operation time; B, blood loss; C, blood transfusion; D, total complication; E, pleural effusion; F, ascites; G, hospital stay. **Supplementary Material 14.** Forest plot of subgroup analysis based on the proportion of patients received major liver resection, the cut value were 40% and 60% for blood loss, while 40% for the reset. A, operation time; B, blood loss; C, blood transfusion; D, total complication; E, hospital stay. **Supplementary Material 15.** Forest plot of subgroup analysis based on the proportion of patients with multiple tumor, the cut value was 25% for operation time and blood transfusion, while 60% for the rest. A, operation time; B, blood loss; C, blood transfusion; D, total complication; E, pleural effusion; F, hospital stay.

## Data Availability

The datasets used and/or analyzed during the current study are available from the corresponding author on reasonable request.
