# Supplementary material for: Effect of intermittent Pringle maneuver on perioperative outcomes and long-term survival following liver resection in patients with hepatocellular carcinoma: a meta-analysis and systemic review
Source: World J Surg Oncol. 2023 Nov 21;21:359. doi: 10.1186/s12957-023-03244-x (PMC10662549; doi:10.1186/s12957-023-03244-x)

Supplementary material 1. Search strategy

| Databases | Search strategy |
| --- | --- |
| Pubmed | #1 ((hepatocellular carcinoma[MeSH Terms]) OR (hepatocellular carcinoma)) OR (HCC)  #2 **((((((blood occlusion) OR (pringle maneuver)) OR (PM)) OR (IPM)) OR (hepatic pedicle clamp)) OR (inflow occlusion)) OR (hilar clamping)**  **#3 ((((((hepatectomy[MeSH Terms]) ) OR (liver resection)) OR (hepatic resection)) OR (surgical resection)) OR (hepatectomy)**  **#4** **#1 AND #2 AND #3** |
| Embase | **#1 'liver cell carcinoma'/exp OR 'hepatocellular carcinoma':ti,ab,kw OR hcc:ti,ab,kw**  **#2 'hepatectomy'/exp OR 'liver resection':ti,ab,kw OR 'hepatic resection':ti,ab,kw OR hepatectomy:ti,ab,kw OR 'surgical resection':ti,ab,kw**  **#3 'blood occlusion':ti,ab,kw OR 'pringle maneuver':ti,ab,kw OR 'hepatic pedicle clamp':ti,ab,kw OR 'inflow occlusion':ti,ab,kw OR pm:ti,ab,kw OR ipm:ti,ab,kw OR 'hilar clamping':ti,ab,kw**  **#4 #1 AND #2 AND #3** |
| Web of Science | #1 hepatocellular carcinoma (Topic) or hcc (Topic)  #2 blood occlusion (Topic) or pringle maneuver (Topic) or hepatic pedicle clamp (Topic) or inflow occlusion (Topic) or hilar clamping (Topic) or pm (Topic) or ipm (Topic)  #3 hepatectomy (Topic) or liver resection (Topic) or hepatic resection (Topic) or surgical resection (Topic)  #4 **#1 AND #2 AND #3** |
| Cochrane Library | #1 MeSH descriptor:[Carcinoma, Hepatocellular] explode all trees OR (hepatocellular carcinoma) :ti,ab,kw OR (hcc) :ti,ab,kw  #2 MeSH descriptor:[Hepatectomy] explode all trees OR (liver resection) :ti,ab,kw OR (hepatic resection) :ti,ab,kw OR (hepatectomy) :ti,ab,kw OR (surgical resection) :ti,ab,kw  #3 (blood occlusion) :ti,ab,kw OR (pringle maneuver) :ti,ab,kw OR (hepatic pedicle clamp) :ti,ab,kw OR (inflow occlusion) :ti,ab,kw OR (hilar clamping) :ti,ab,kw OR (pm) :ti,ab,kw OR (ipm) :ti,ab,kw  #4 #1 AND #2 AND #3 |

Supplementary material 2. Risk assessment of RCT

| Study | Random sequence generation | Allocation concealment | Blinding of outcome assessment | Blinding of participants and personnel | Incomplete outcome data | Selective reporting | Other bias |
| --- | --- | --- | --- | --- | --- | --- | --- |
| Lee 2019 | Low Risk | Low Risk | Unclear | Low Risk | Low Risk | Low Risk | Low Risk |

Supplementary material 3. NOS score of non-RCT studies

| Study | Selection | | | | Comparability | Outcome | | | Total score |
| --- | --- | --- | --- | --- | --- | --- | --- | --- | --- |
|  | Representativeness  of the  exposed cohort | Selection of  the non- exposed cohort | Ascertainment  of exposure | Demonstration  of outcome |  | Assessment of outcome | Follow-up was long enough | Adequacy  of follow up |  |
| Xia | * | * | * | * | * | * | * | * | 8 |
| Huang JW | * | * | * | * |  | * | * | * | 7 |
| Huang ZP | * | * | * | * | ** | * | * | * | 9 |
| Hao | * | * | * | * | ** | * |  | * | 8 |
| Hao | * | * | * | * | ** | * | * | * | 9 |
| Famularo | * | * | * | * | * | * | * | * | 8 |
| Wei | * | * | * | * | * | * | * | * | 9 |
| Galindo | * | * | * | * | * | * | * | * | 9 |
|  |  |  |  |  |  |  |  |  |  |

Supplementary material 4. Subgroup analysis for overall survival and disease-free survival.

A, overall survival; B, disease-free survival.


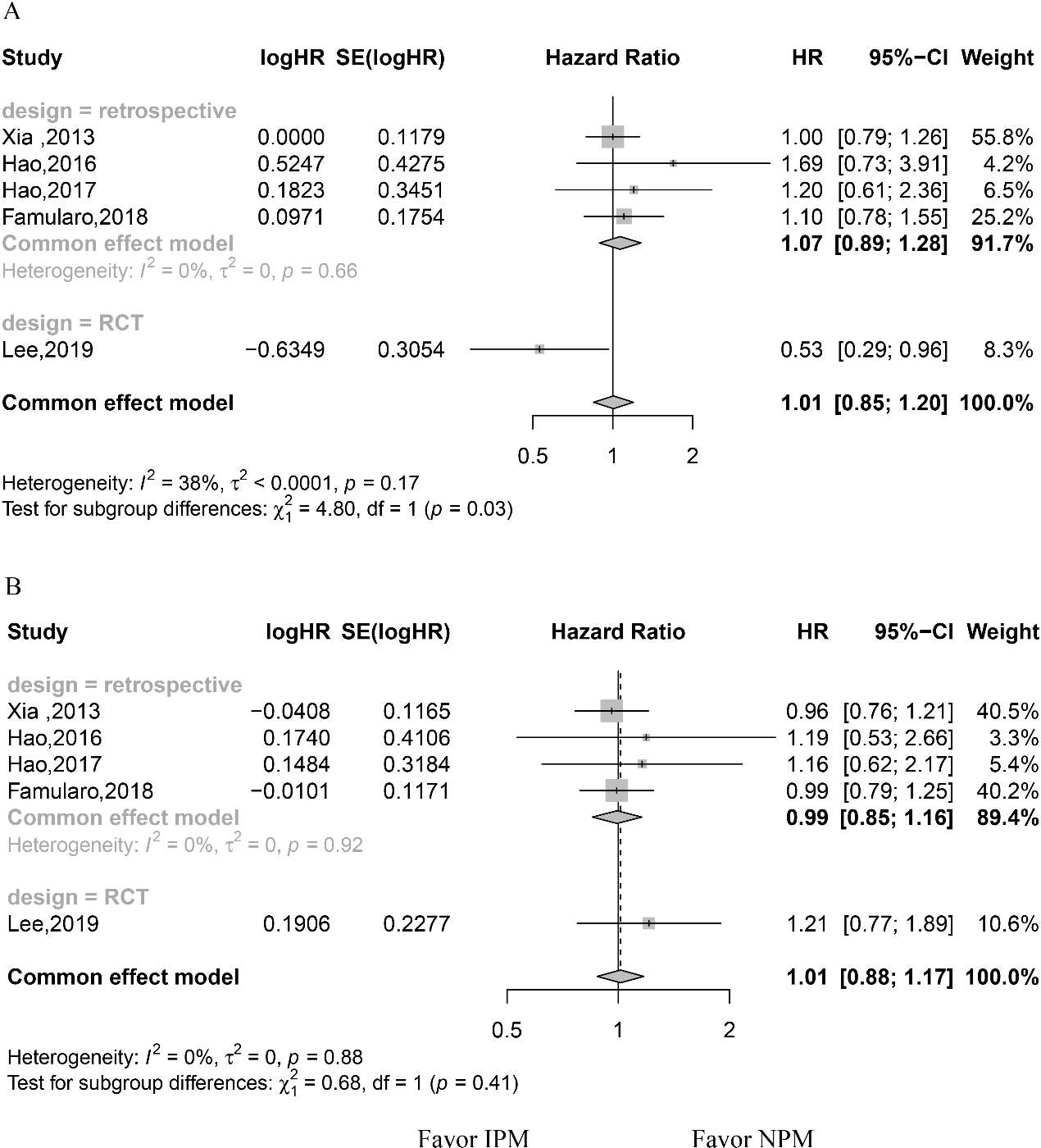


Supplementary material 5. Sensitivity analyses for overall survival and disease-free survival.

A, overall survival; B, disease-free survival.


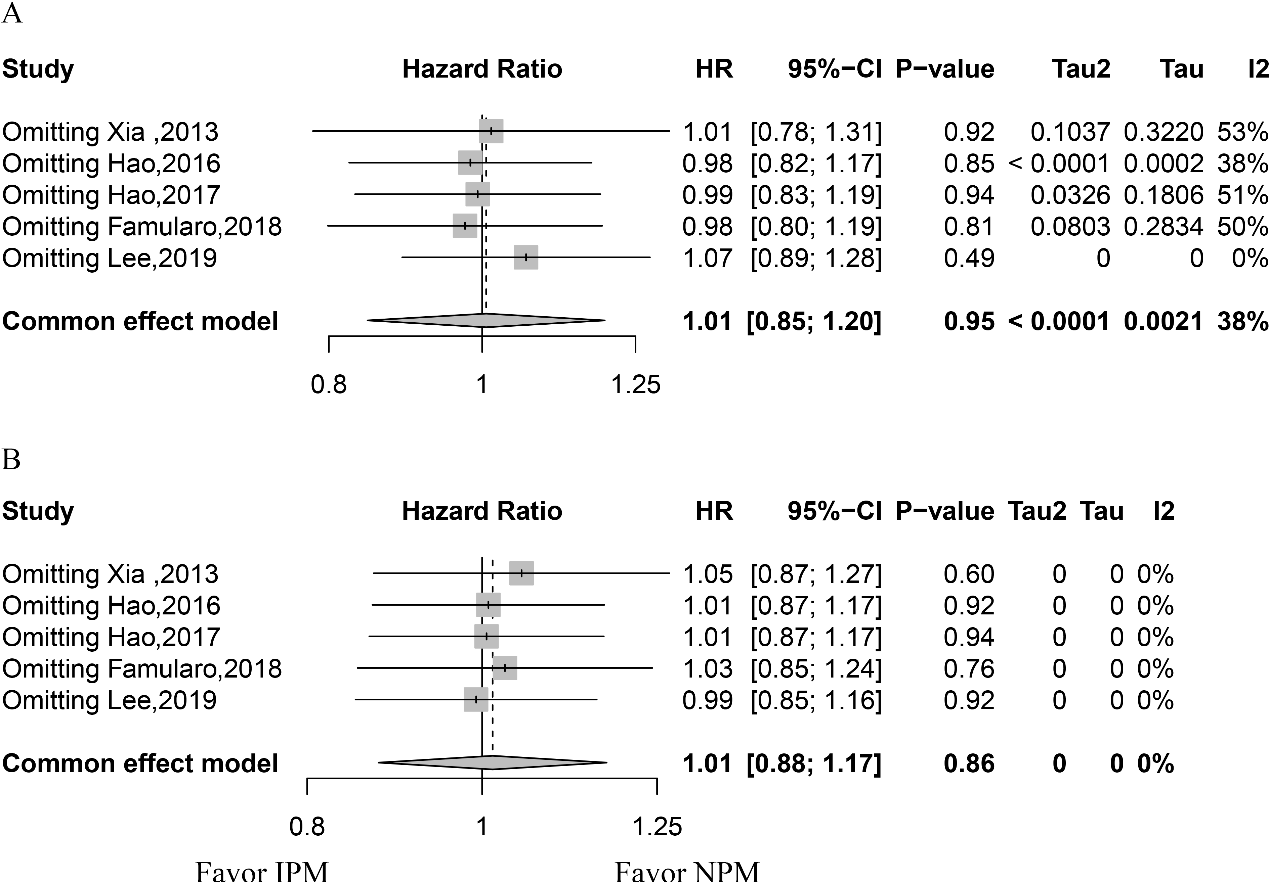


Supplementary material 6. Funnel plot for overall survival and disease-free survival. A, overall survival; B, disease-free survival.
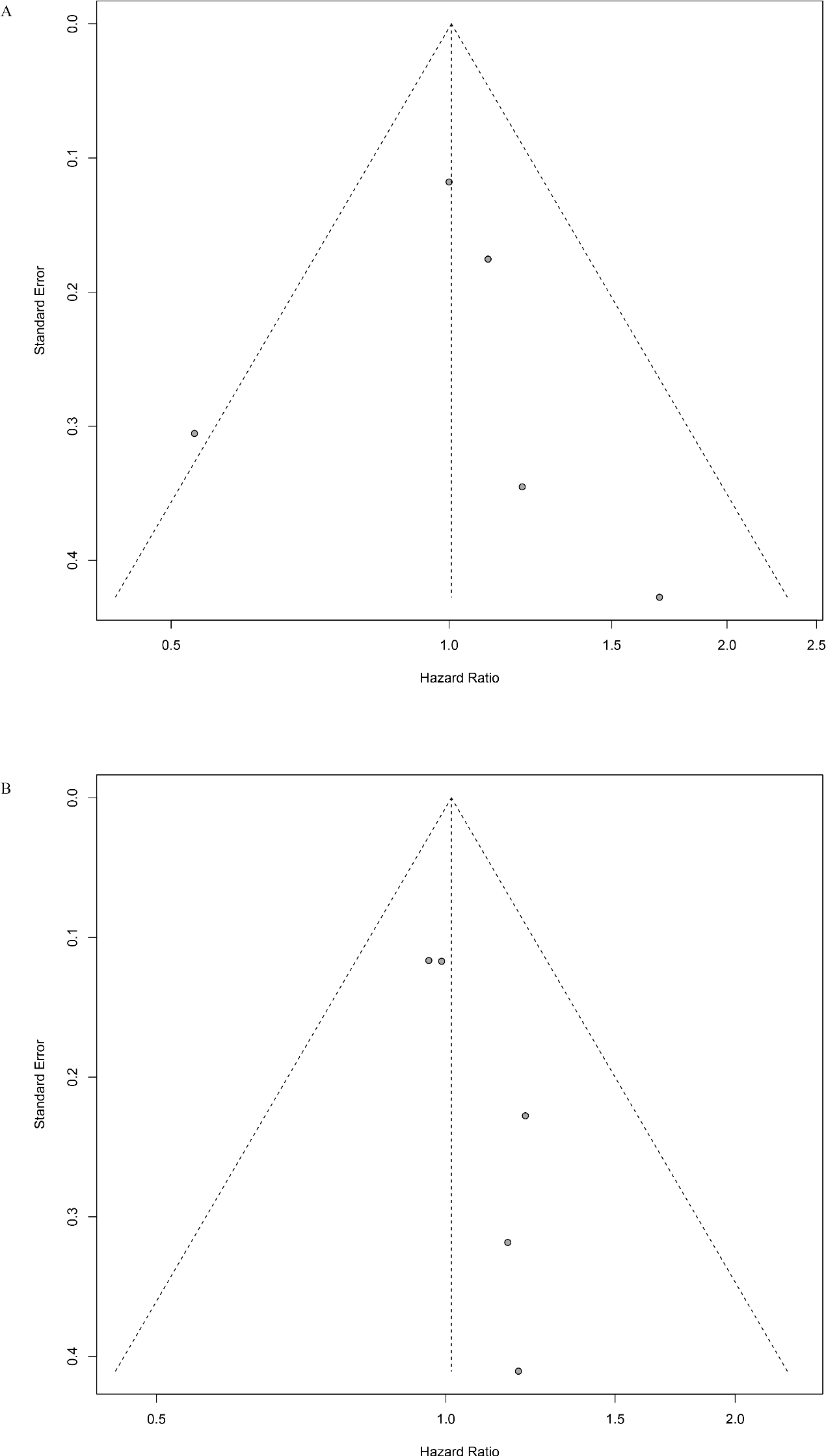


Supplementary material 7. The effect of publication bias evaluated by using the trim and fill method for overall survival and disease-free survival.

A, overall survival; B, disease-free survival.


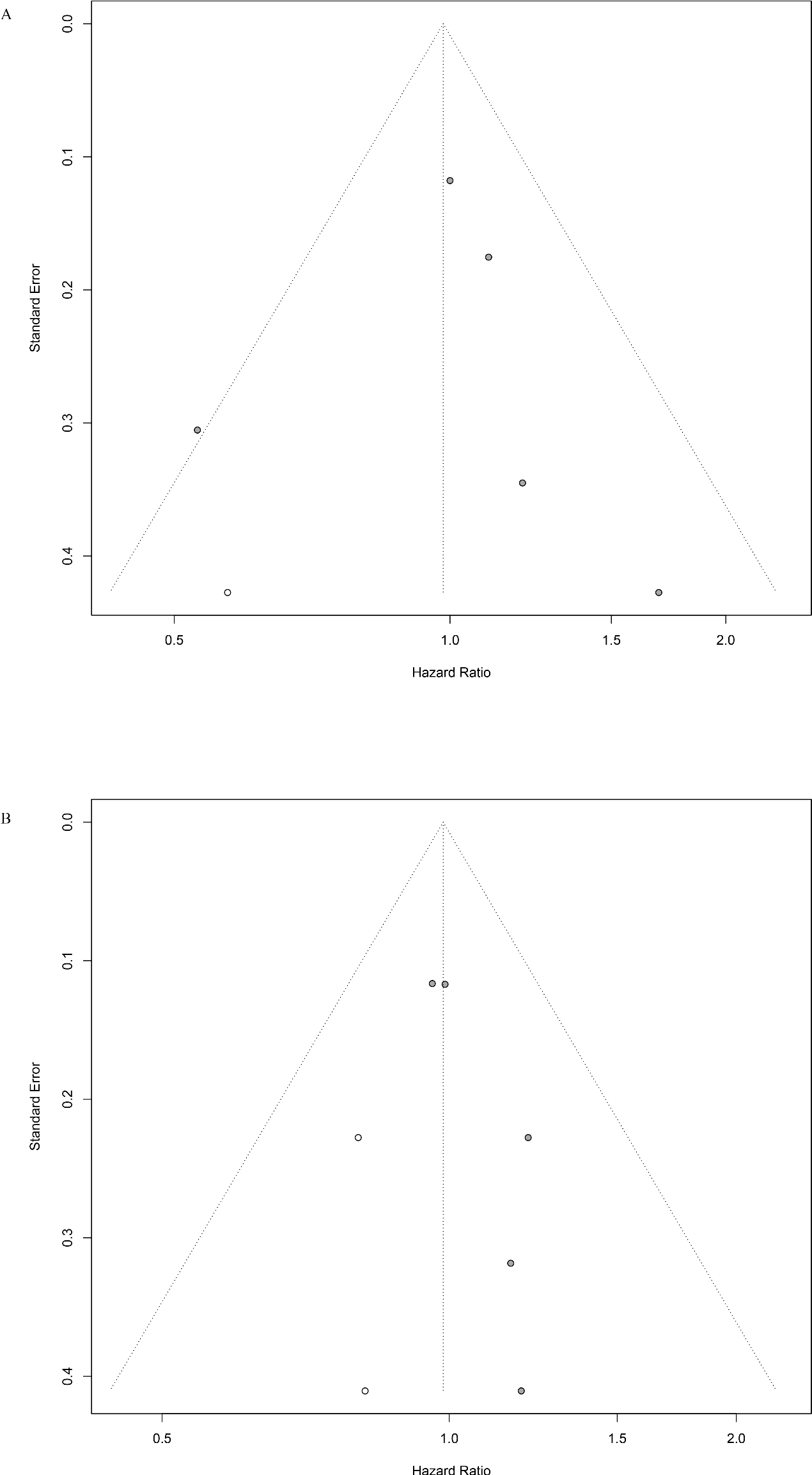


Supplementary material 8. Forest plot after trimming and filling for overall survival and disease-free survival.

A, overall survival; B, disease-free survival.


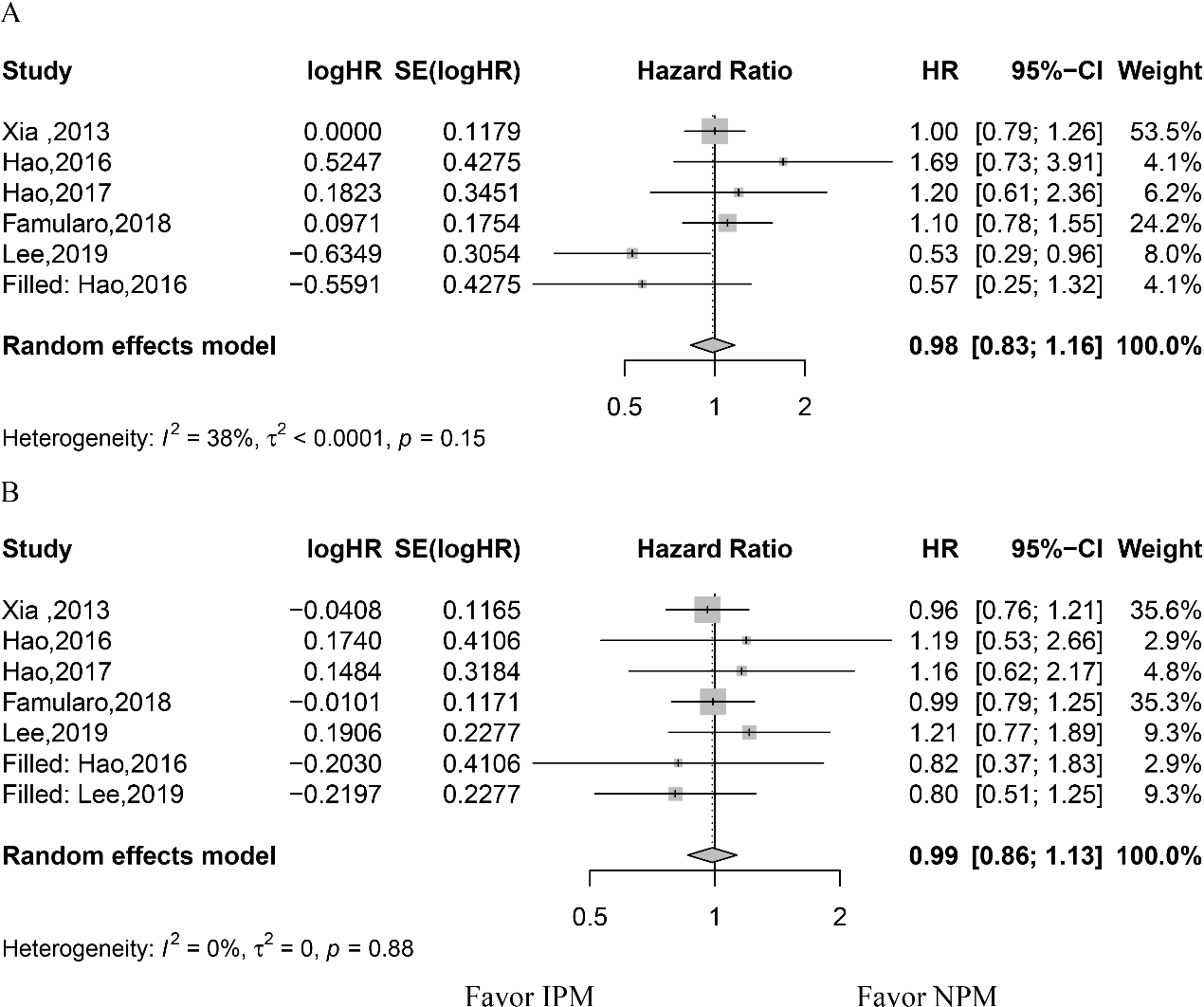


Supplementary material 9. Forest plot of sensitivity analysis for blood loss.


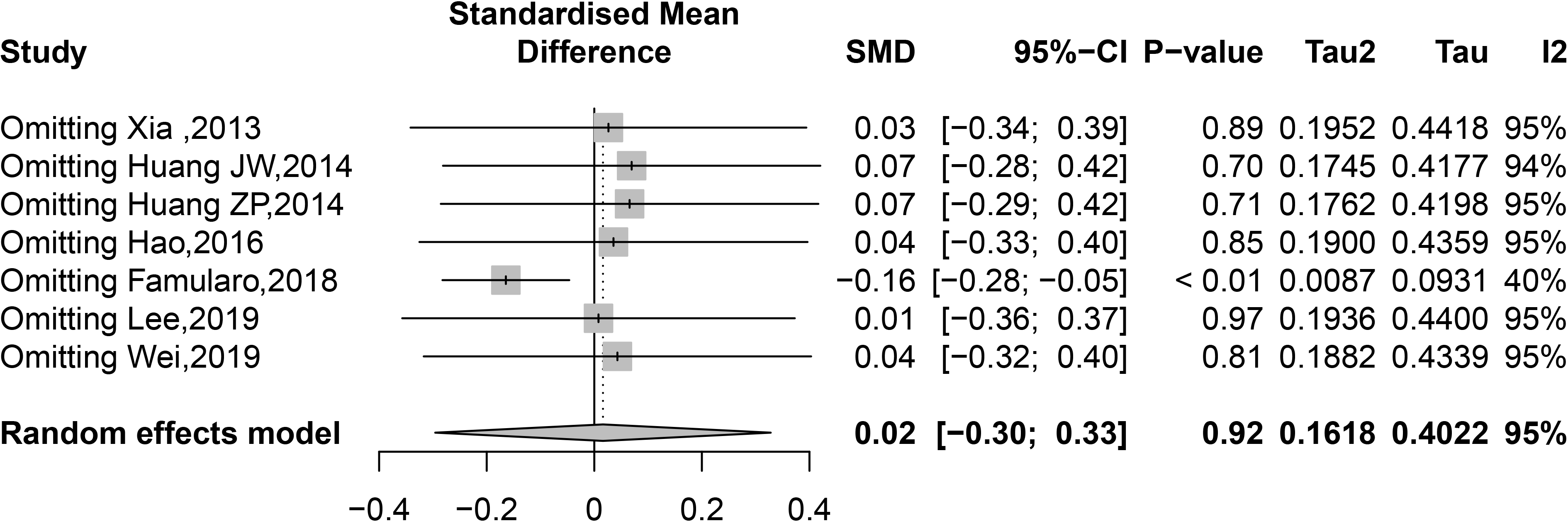


Supplementary material 10. Forest plot for blood loss after omitting the study by Fumularo *et al*.


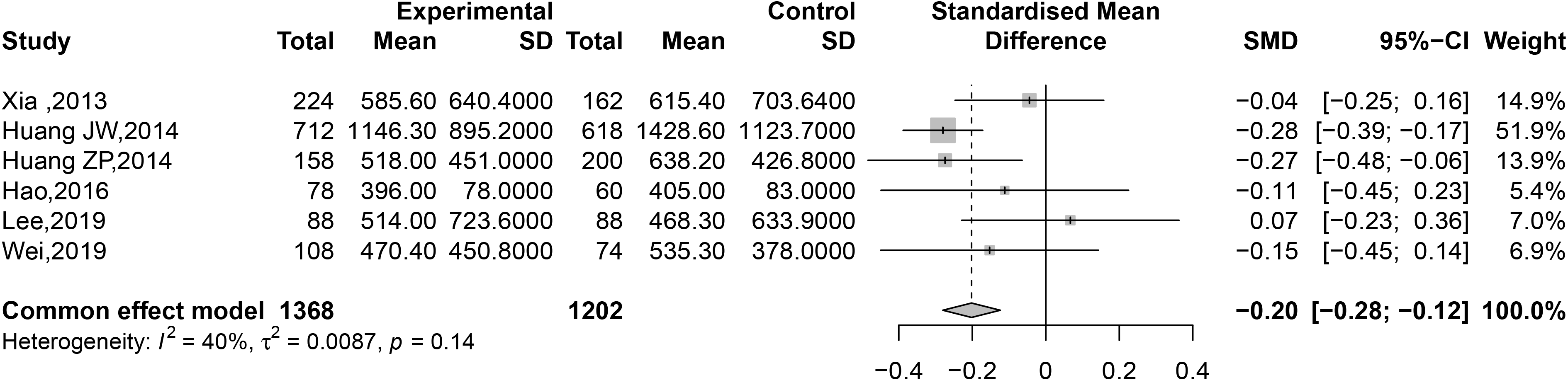


Supplementary material 11. Forest plot of sensitivity analysis for blood loss after omitting the study by Fumularo *et al*.


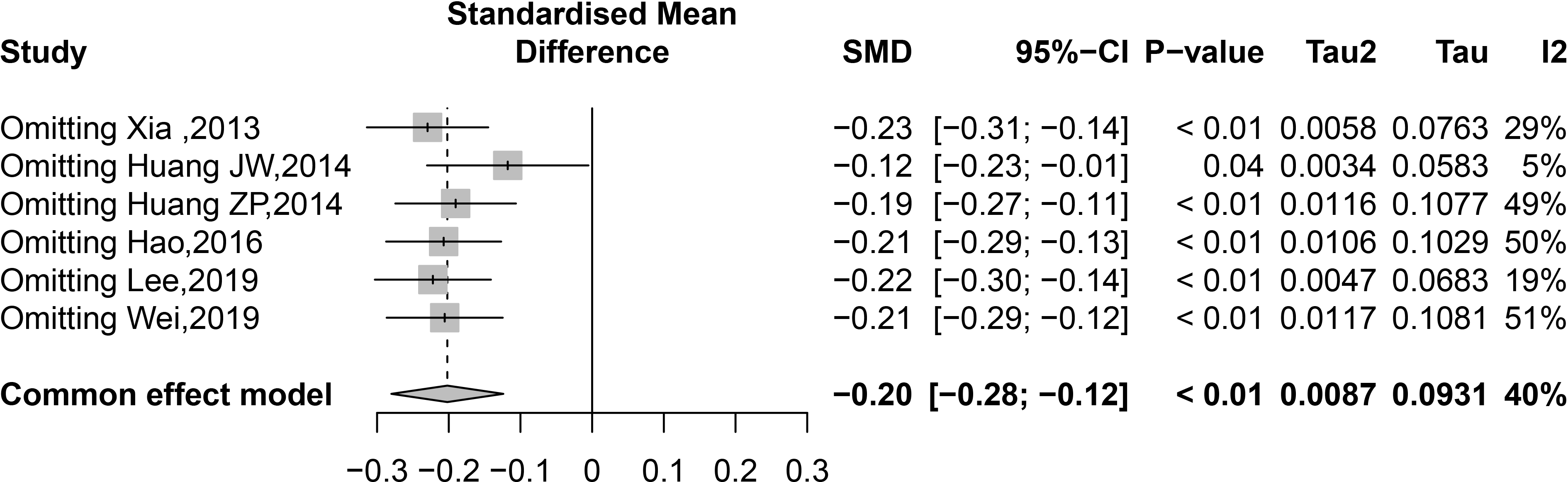


Supplementary material 12. Forest plot of subgroup analysis based on the proportion of patients with Child A, the cut value was 90%. A, operation time; B, blood loss; C, blood transfusion; D, total complication; E, pleural effusion; F, hospital stay.

A


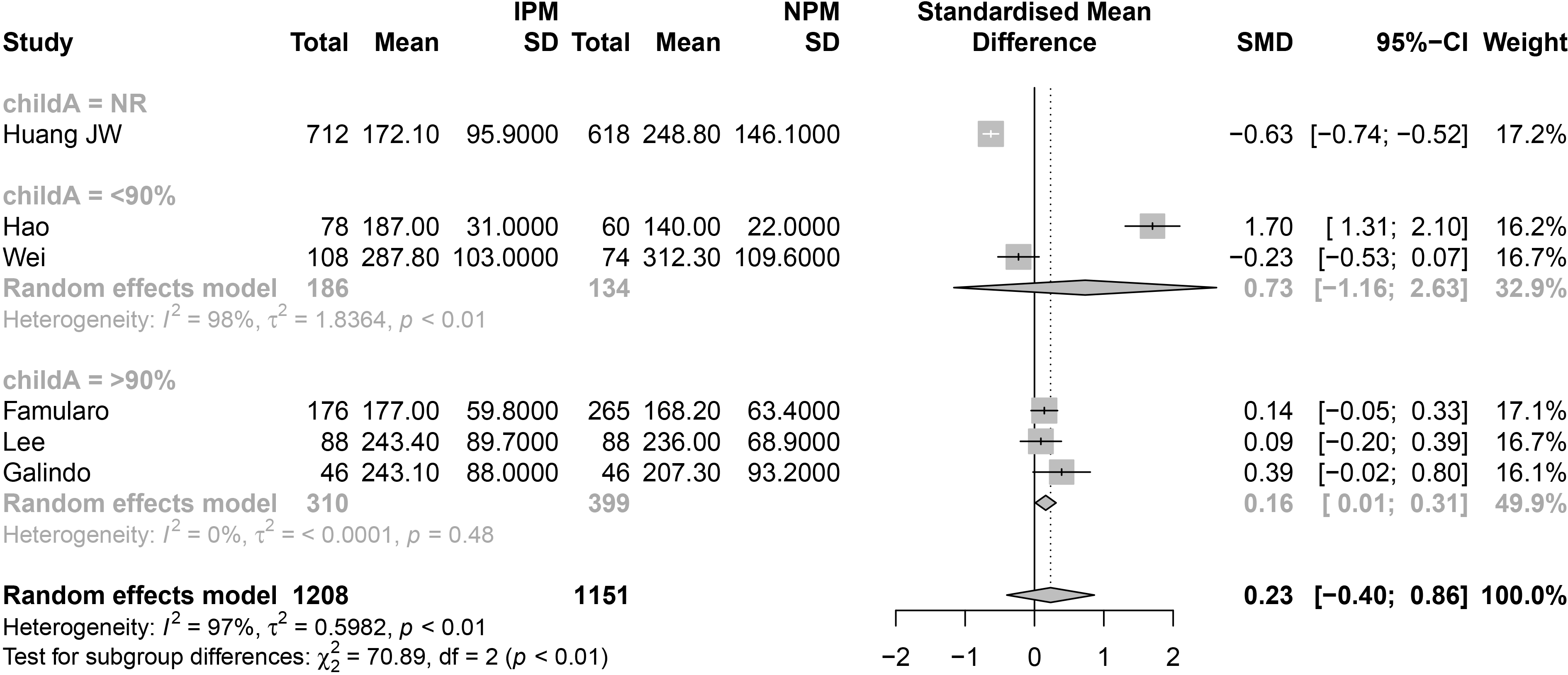


B


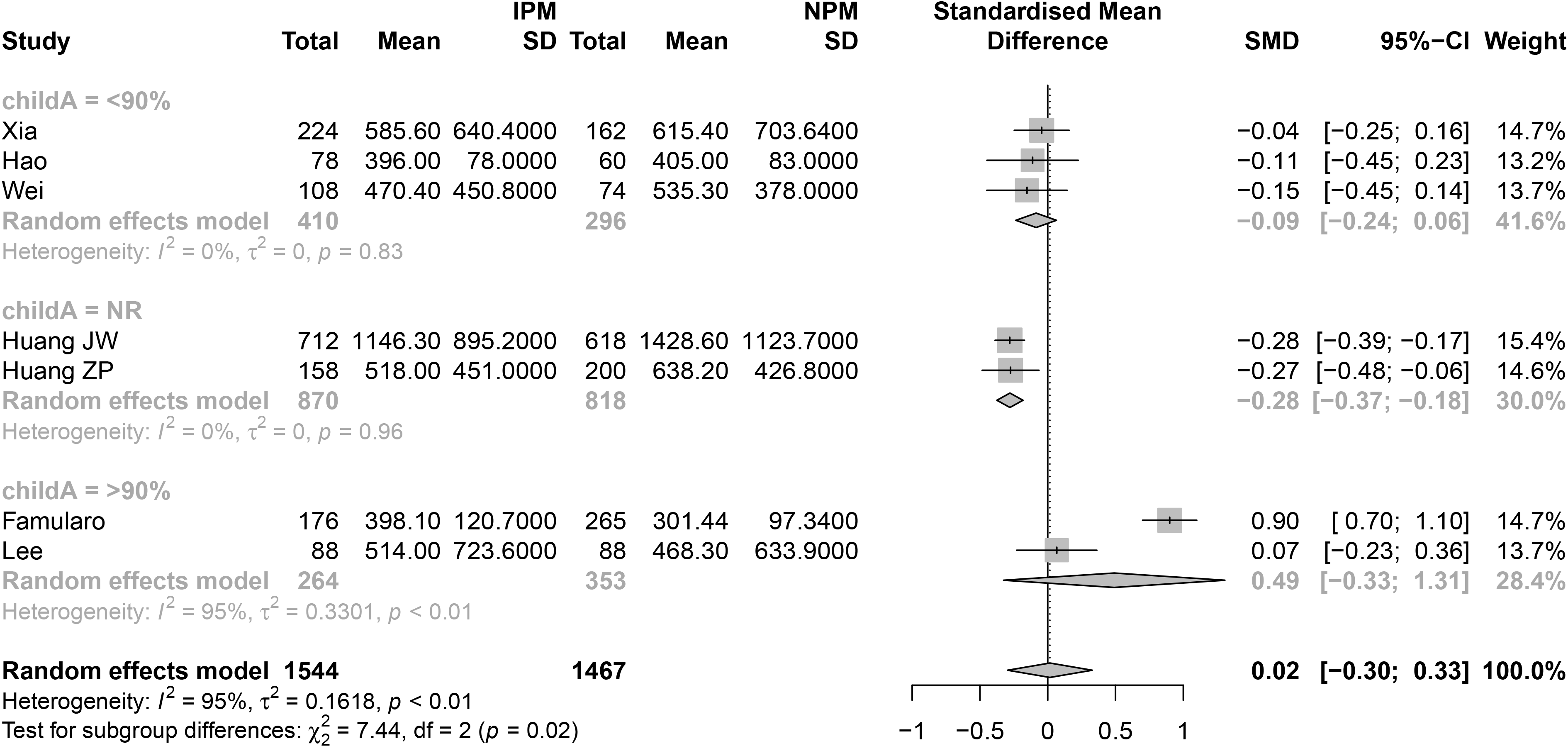


C


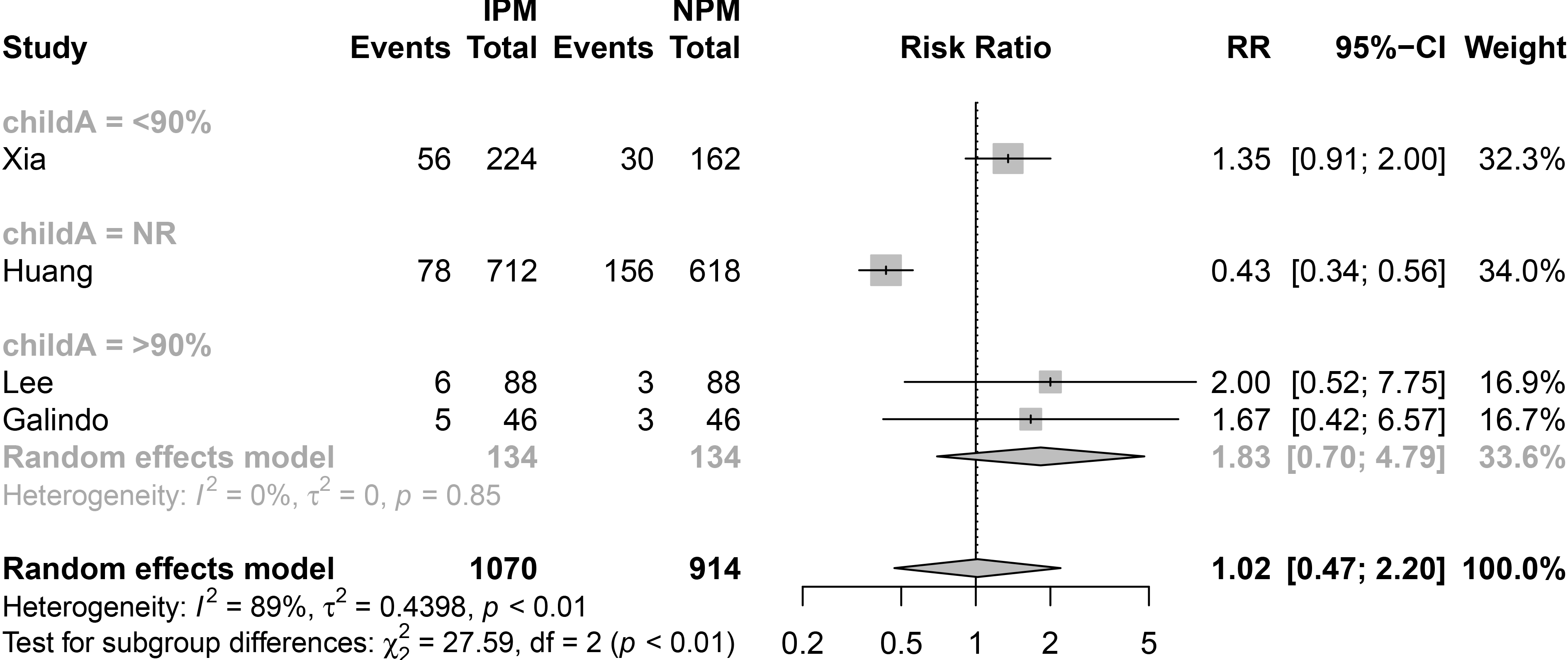


D


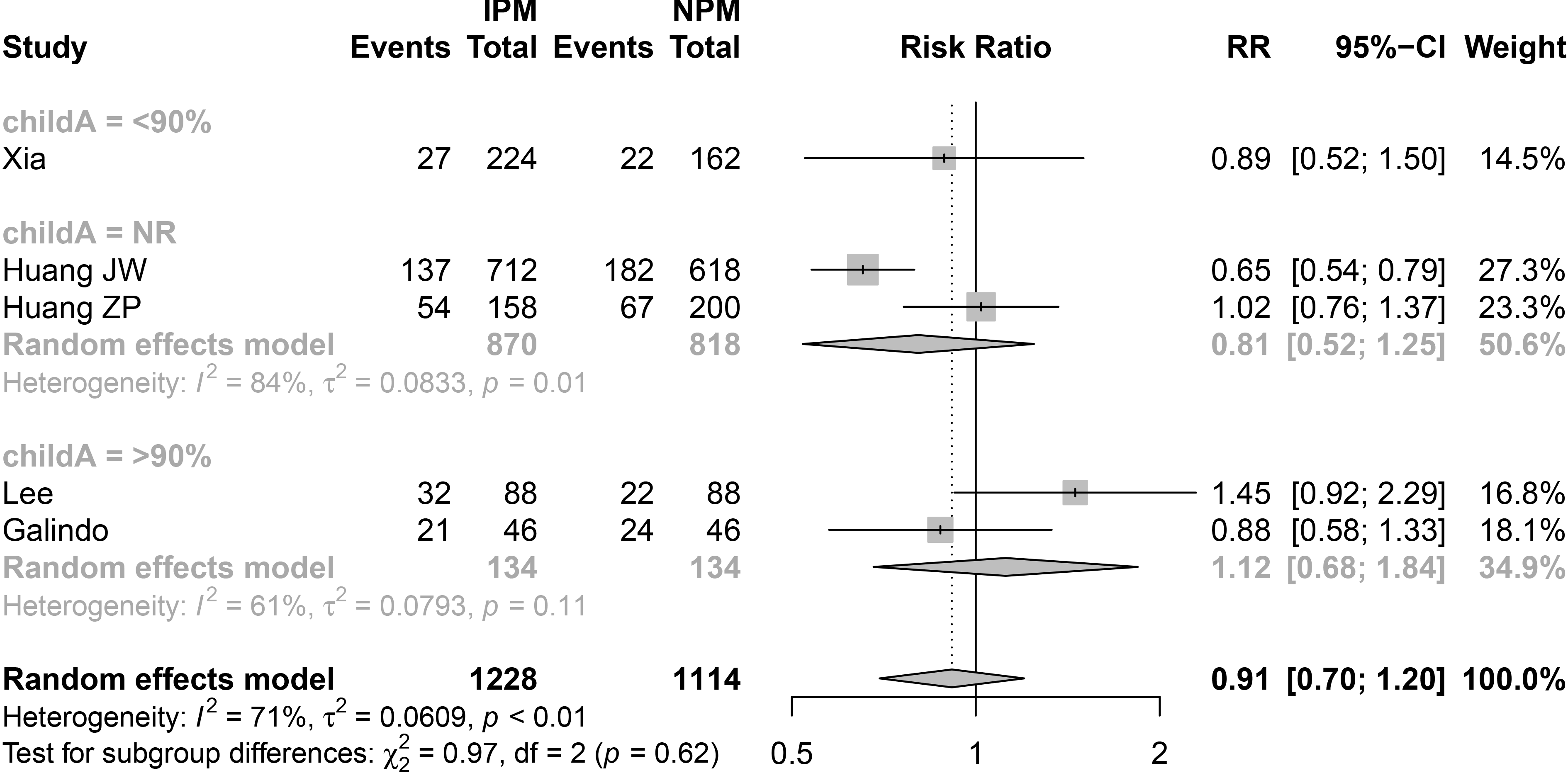


E


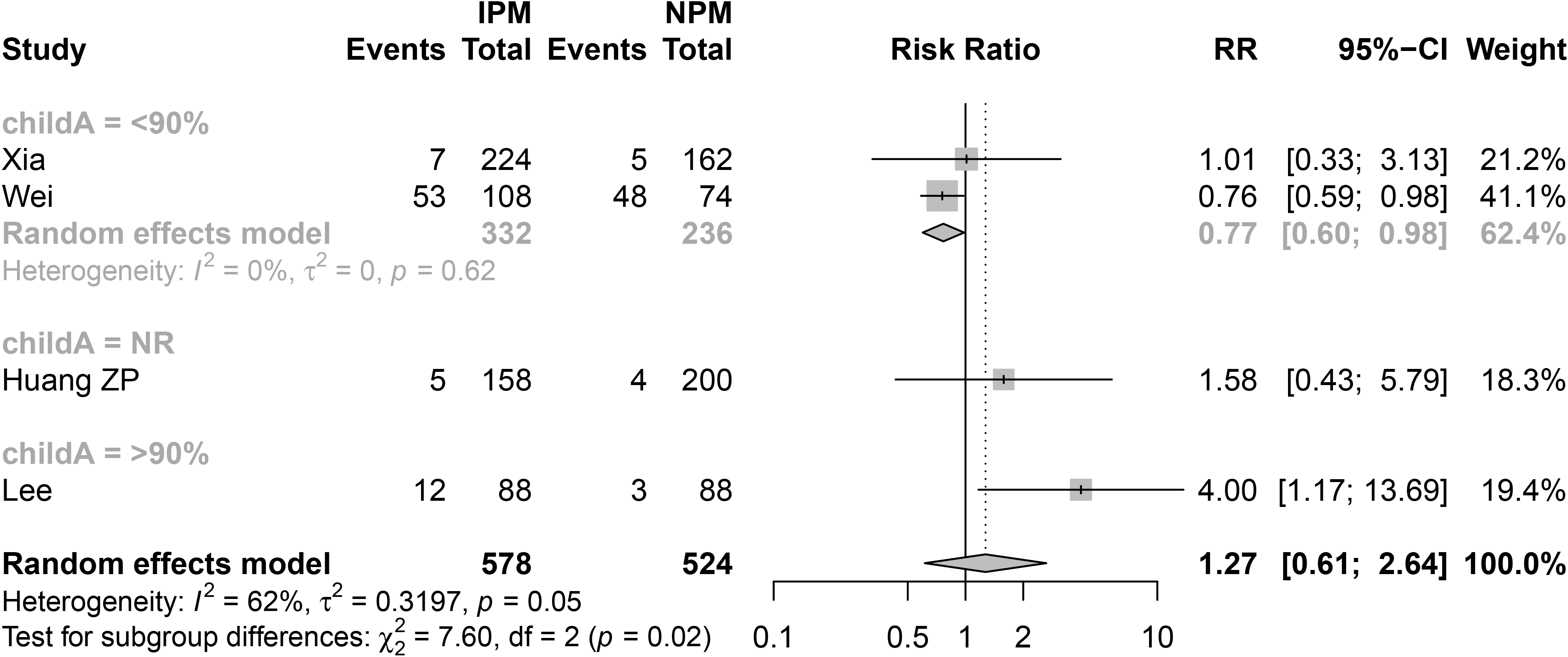


F


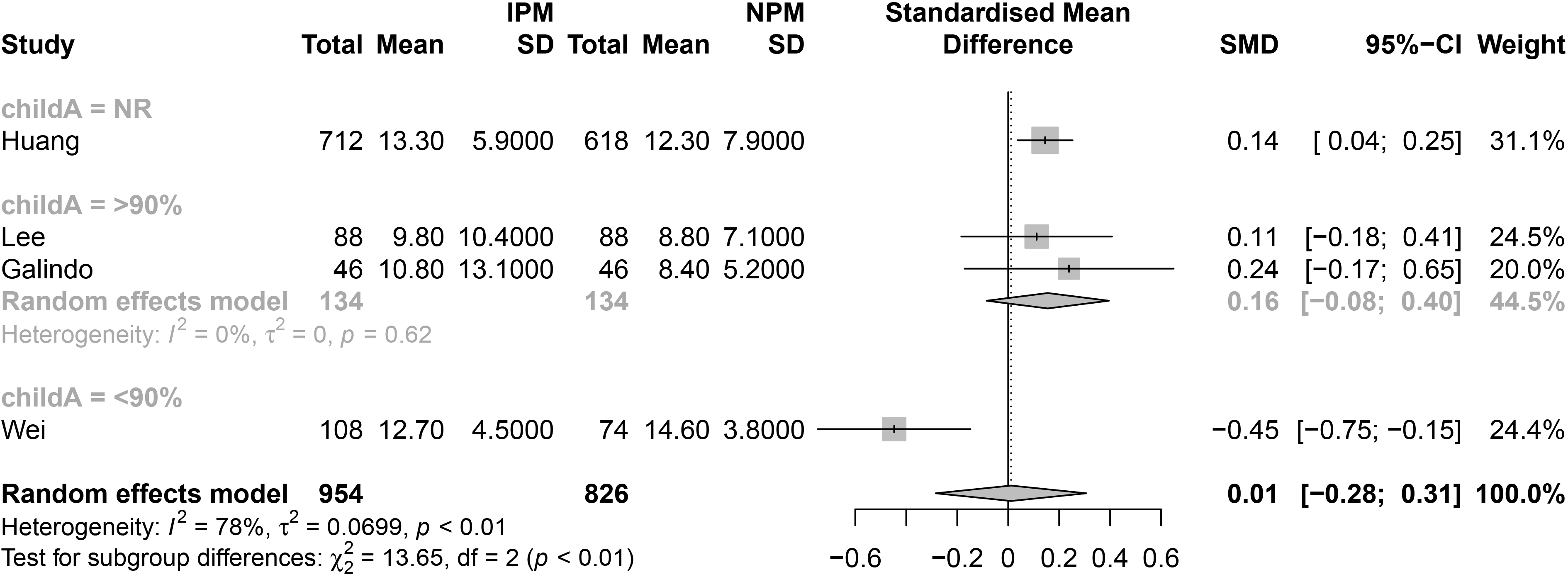


Supplementary material 13. Forest plot of subgroup analysis based on the proportion of patients with liver cirrhosis, the cut value was 70% for operation time and blood transfusion, while 60% for the rest. A, operation time; B, blood loss; C, blood transfusion; D, total complication; E, pleural effusion; F, ascites; G, hospital stay.

A


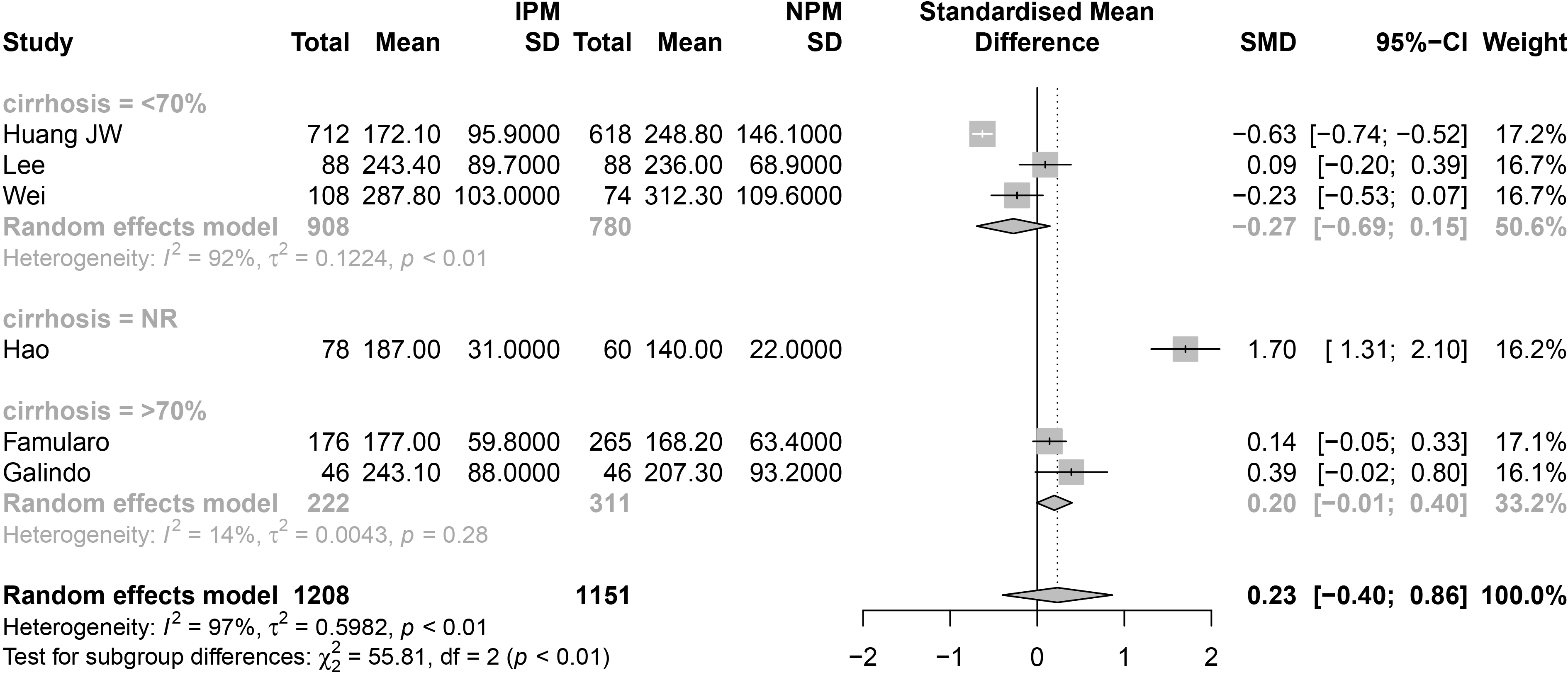


B


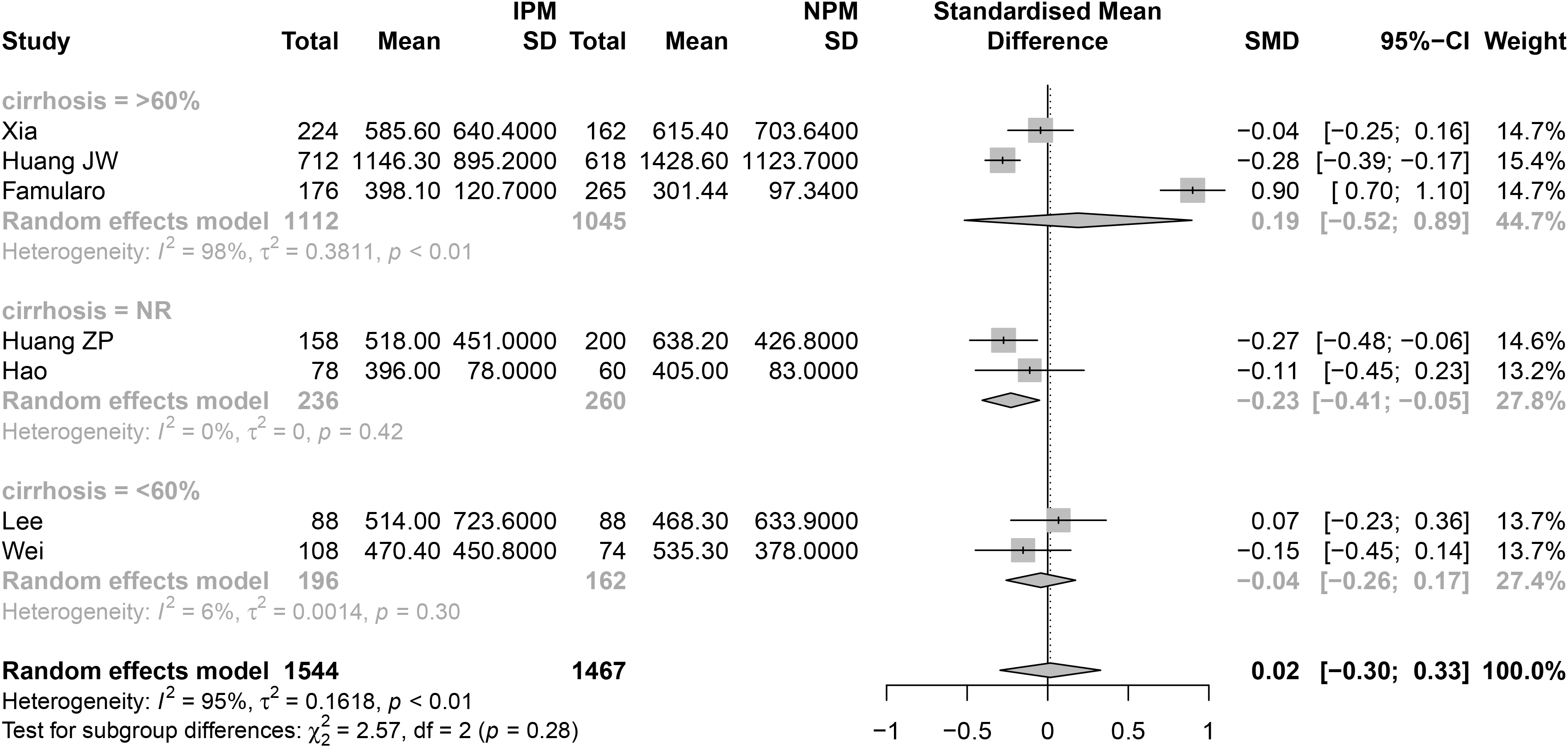


C


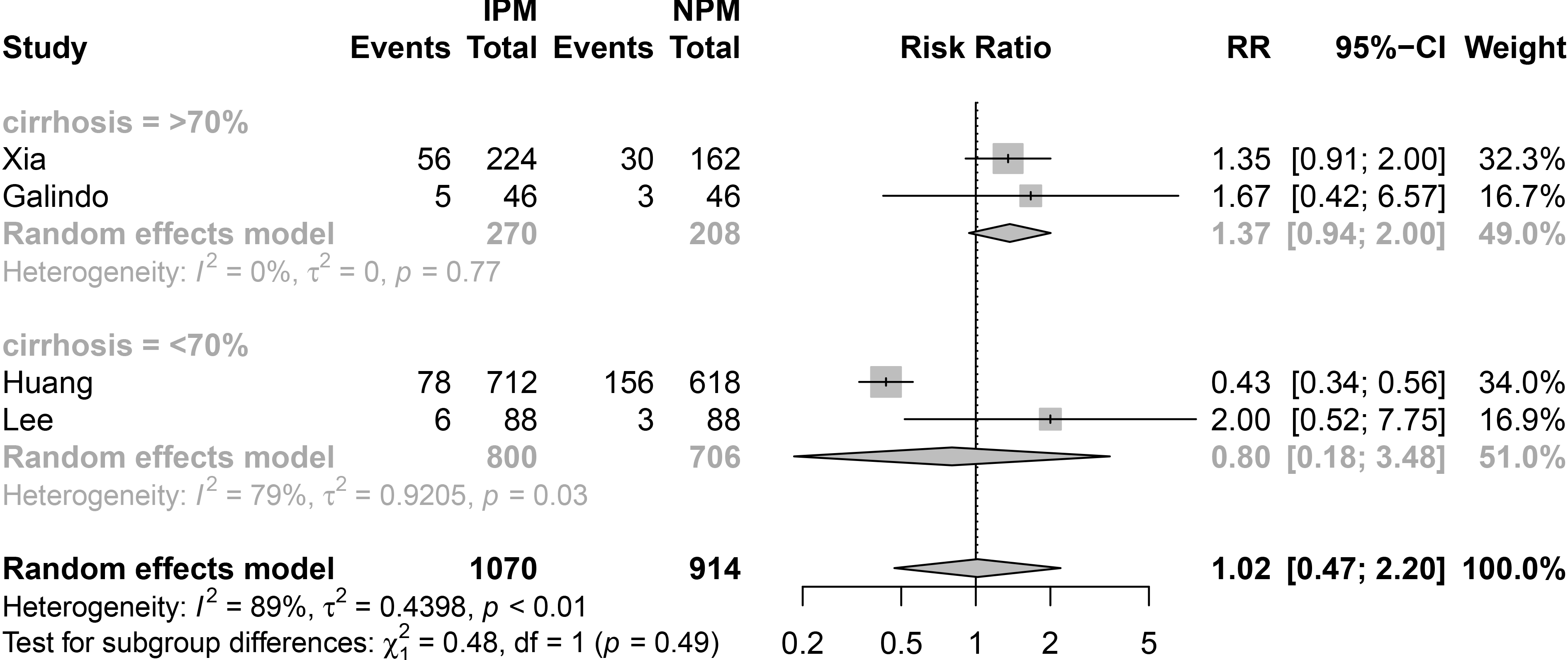


D


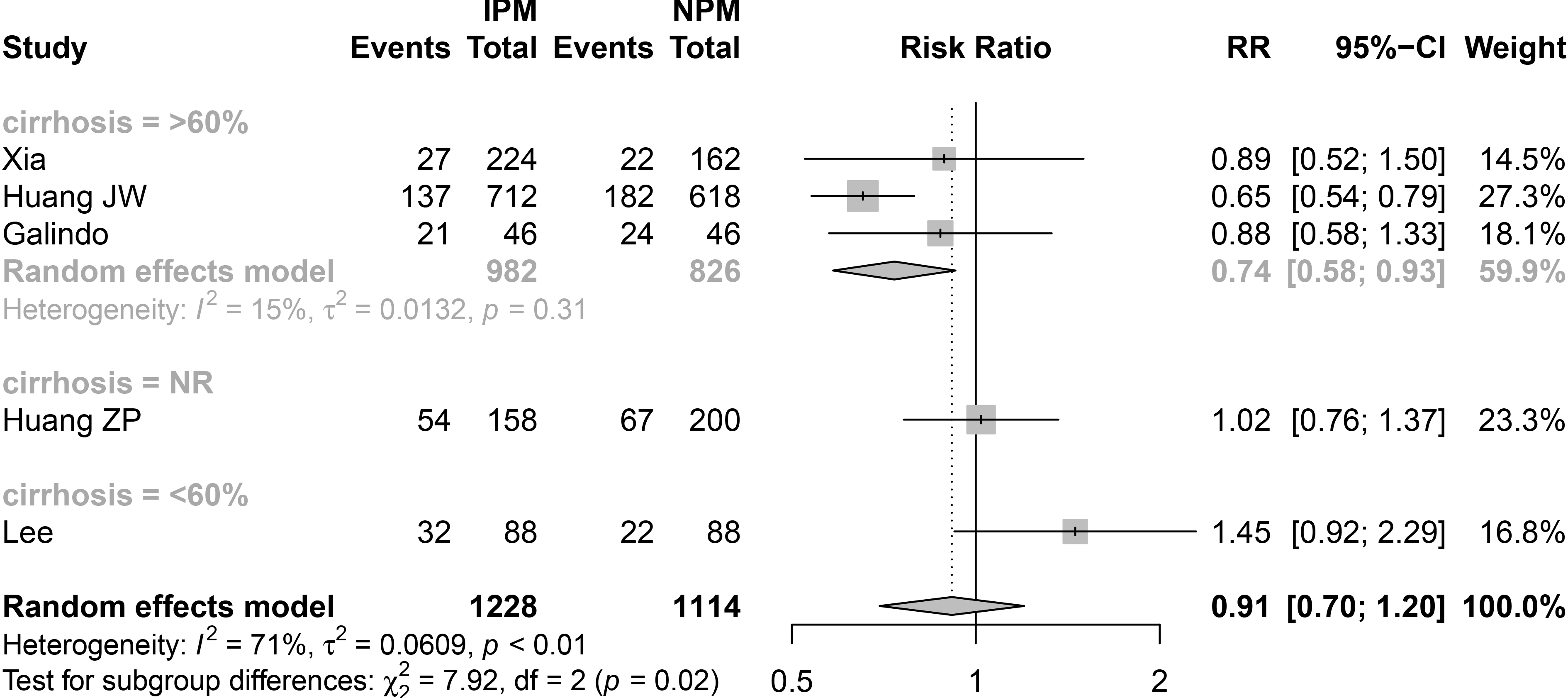


E


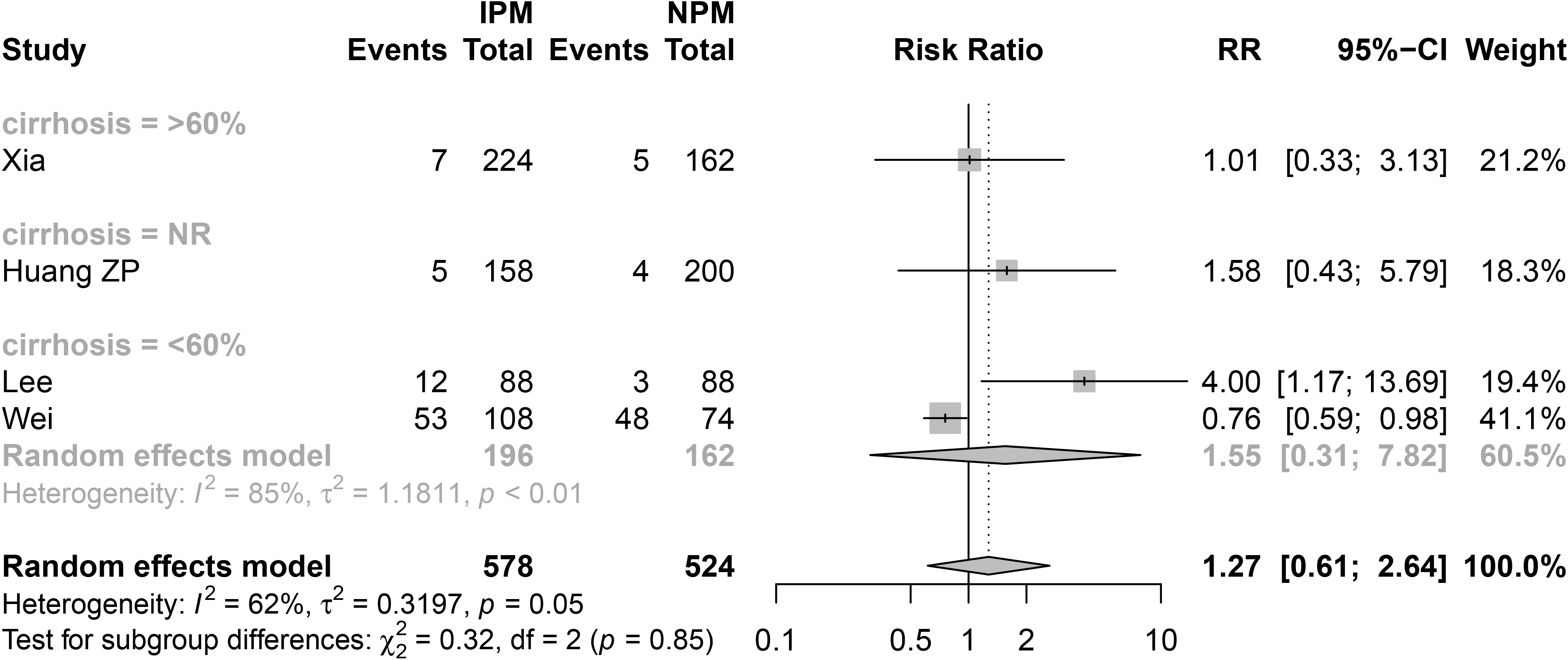


F


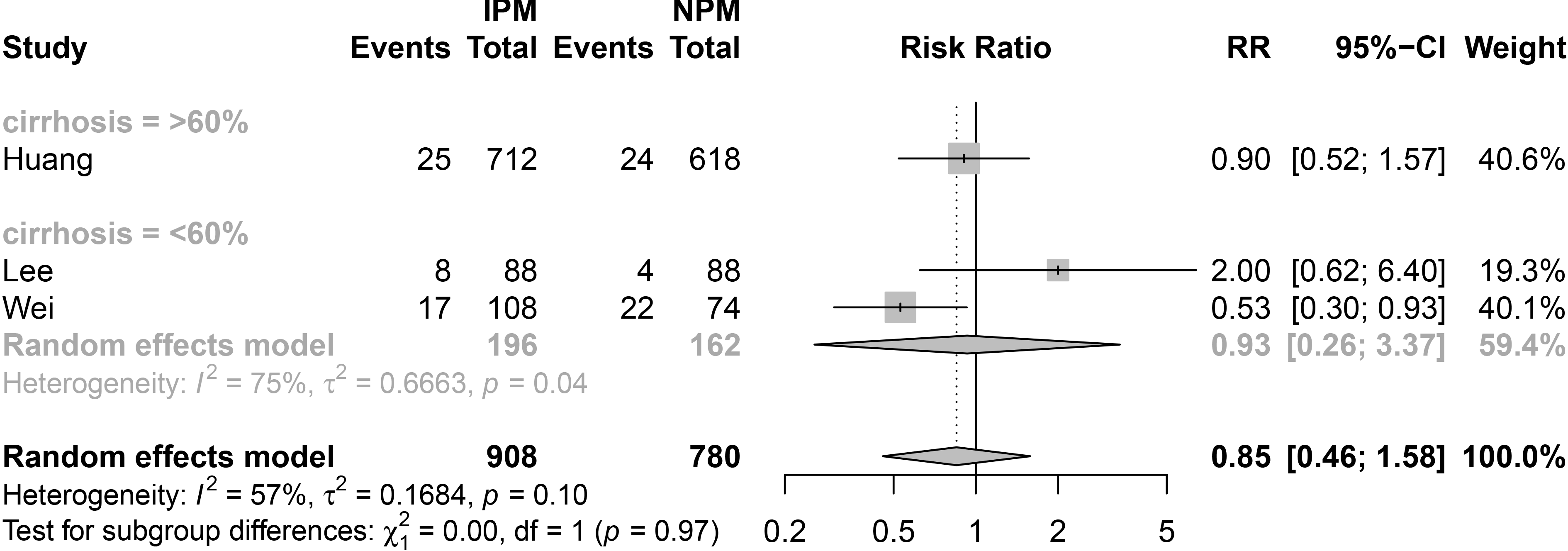


G


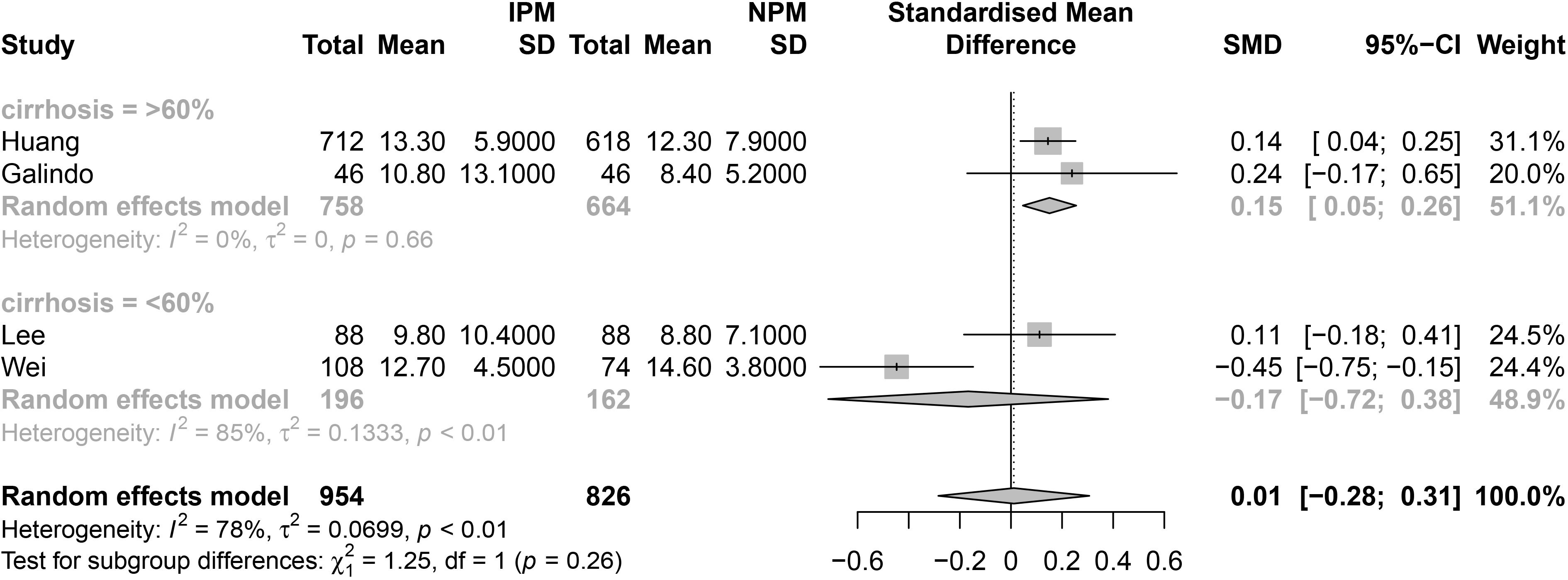


Supplementary material 14. Forest plot of subgroup analysis based on the proportion of patients received major liver resection, the cut value were 40% and 60% for blood loss, while 40% for the reset. A, operation time; B, blood loss; C, blood transfusion; D, total complication; E, hospital stay.

A


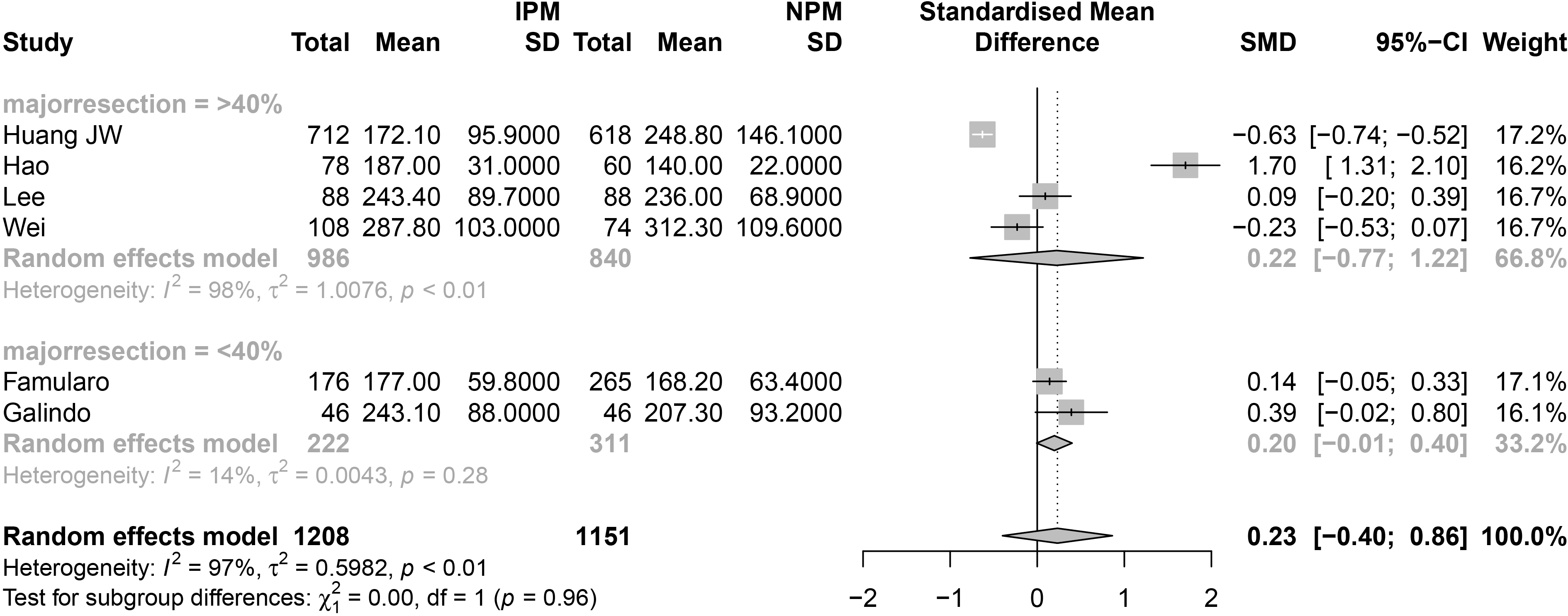


B


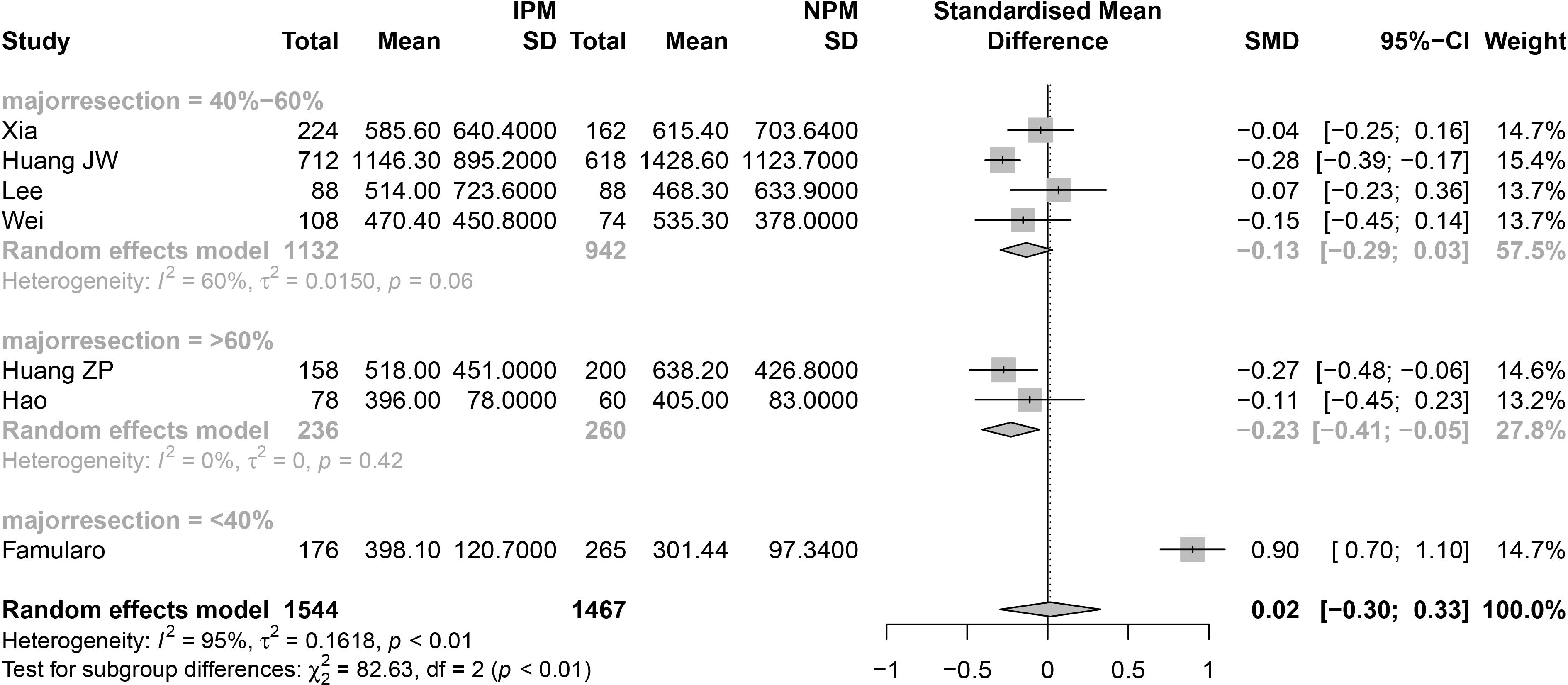


C


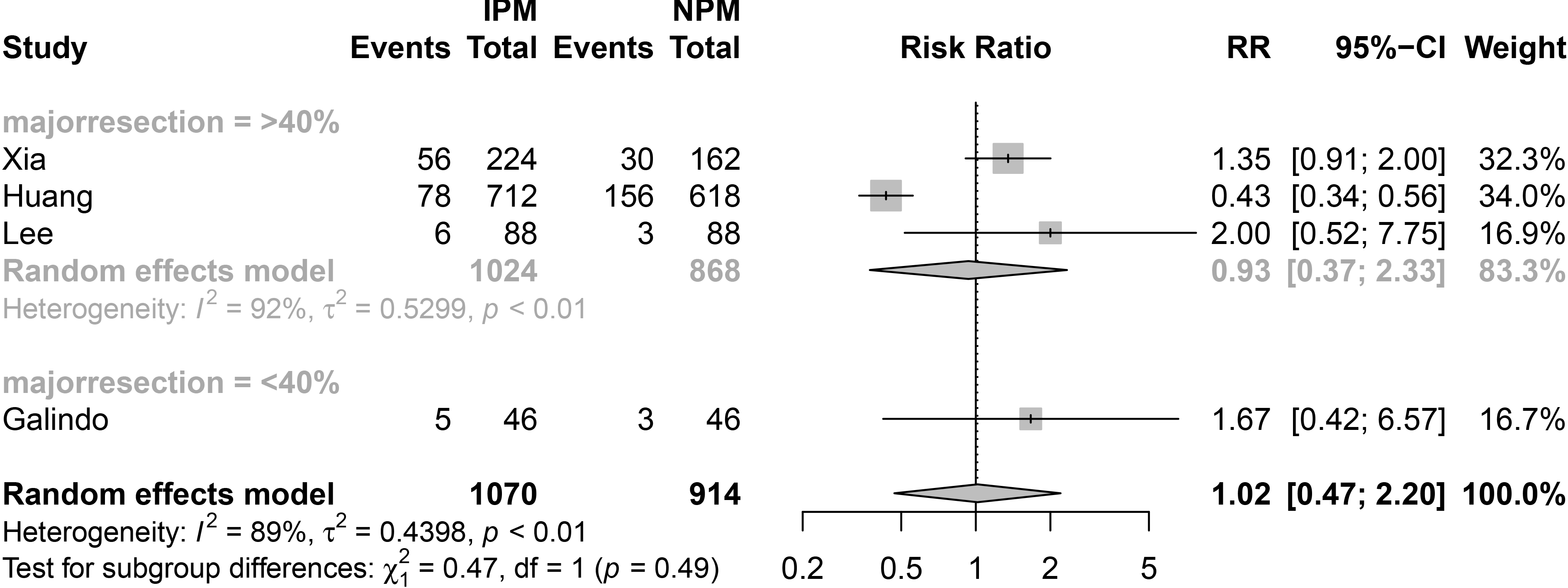


D


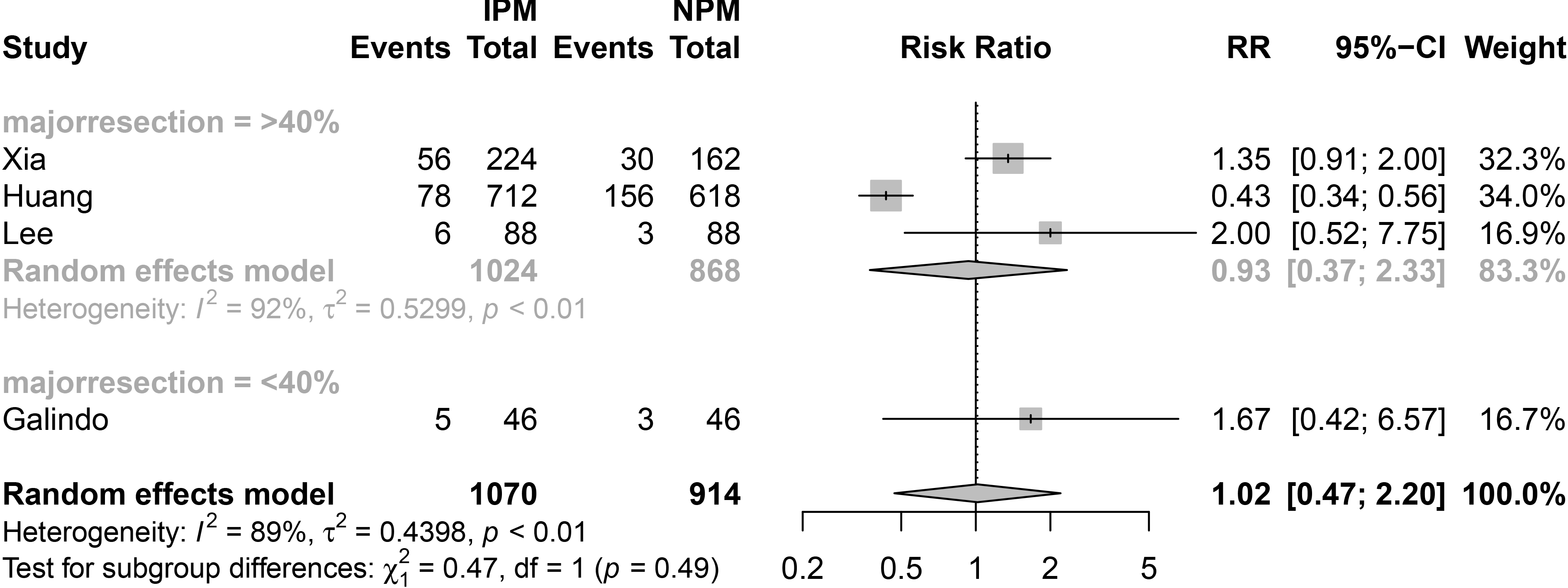


E


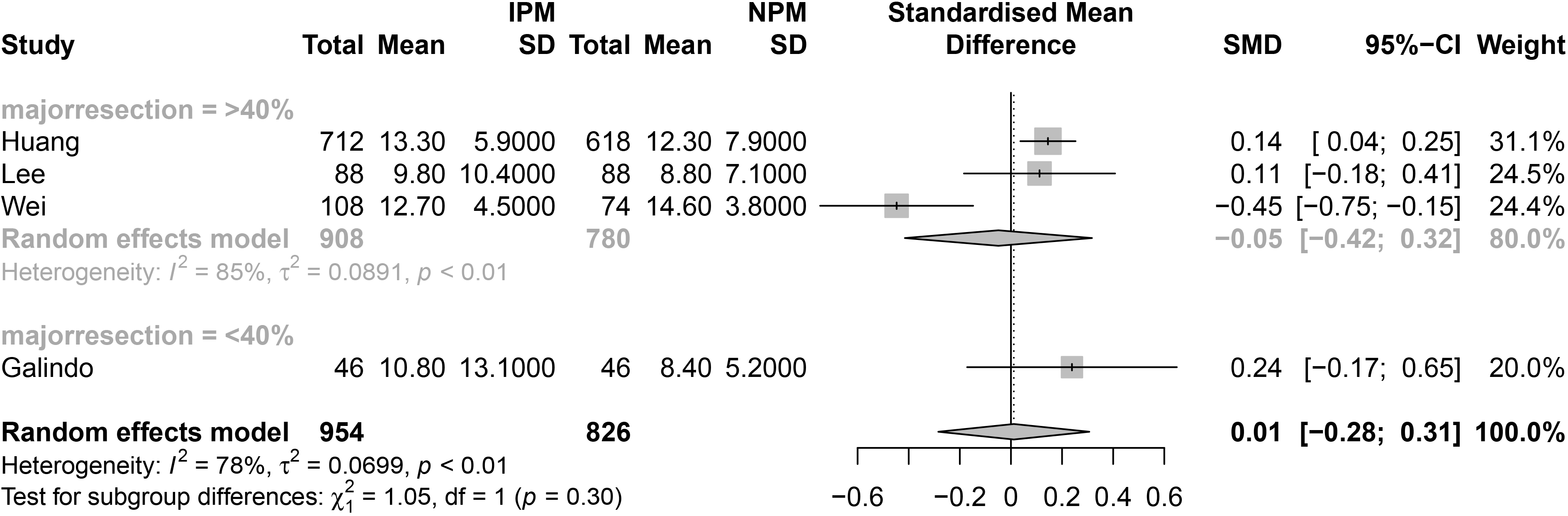


Supplementary material 15. Forest plot of subgroup analysis based on the proportion of patients with multiple tumor, the cut value was 25% for operation time and blood transfusion, while 60% for the rest. A, operation time; B, blood loss; C, blood transfusion; D, total complication; E, pleural effusion; F, hospital stay.

A


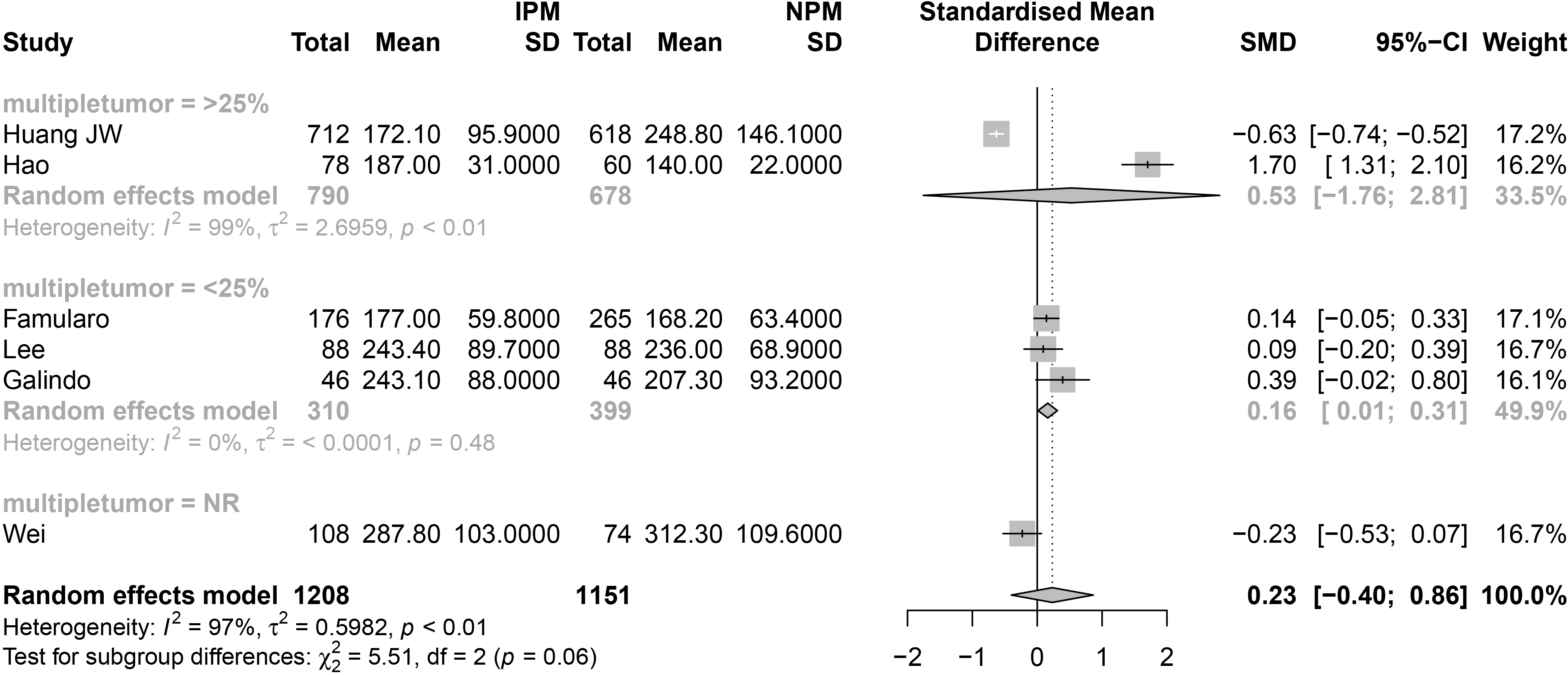


B


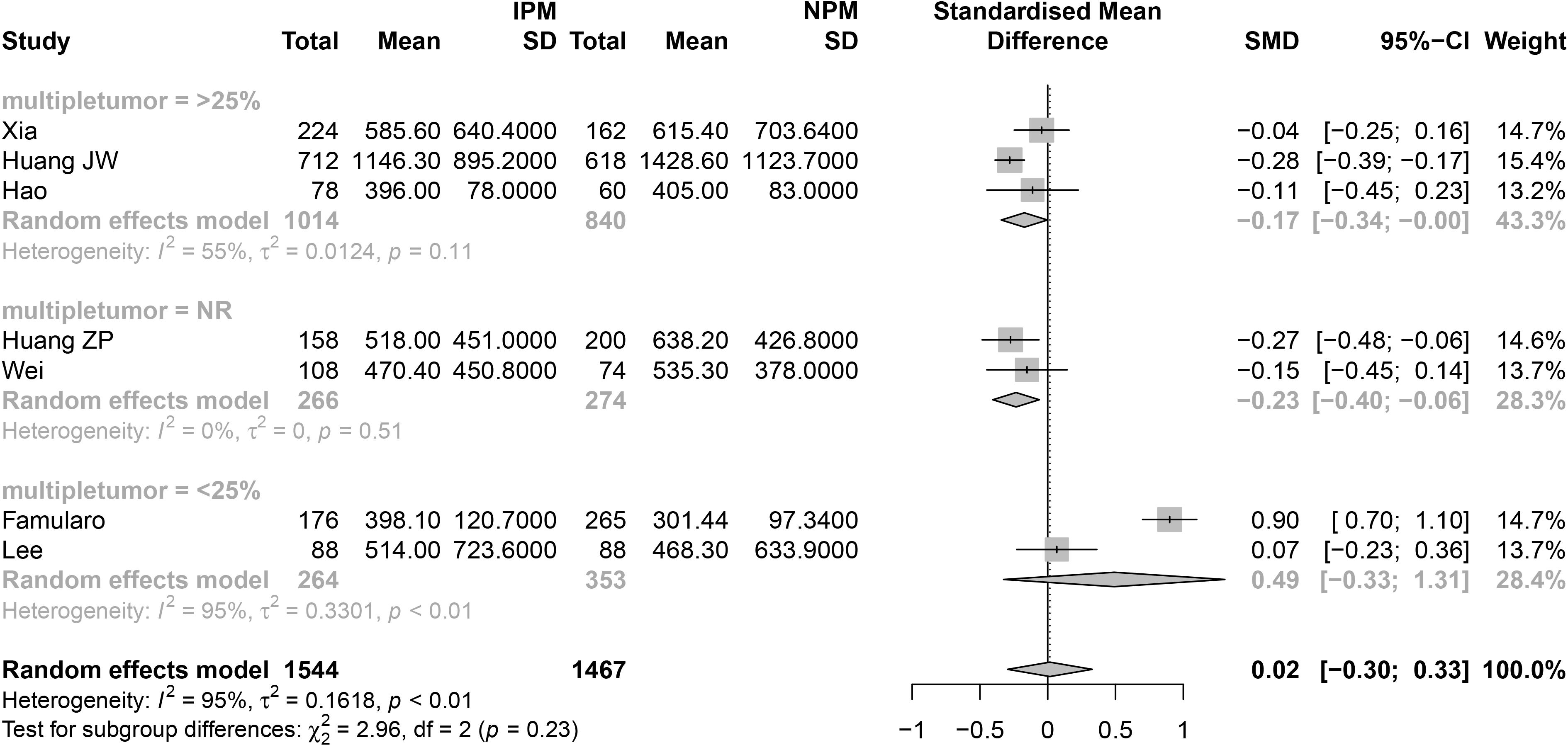


C


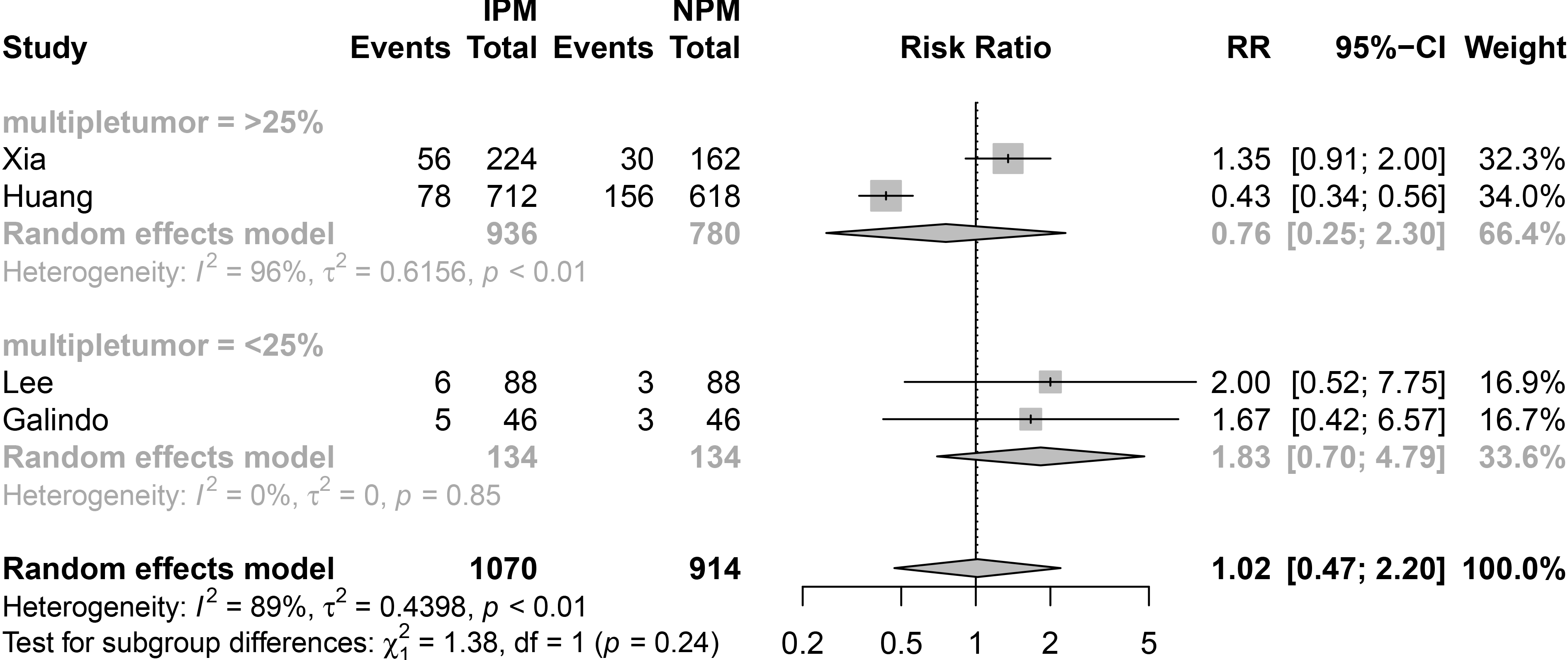


D


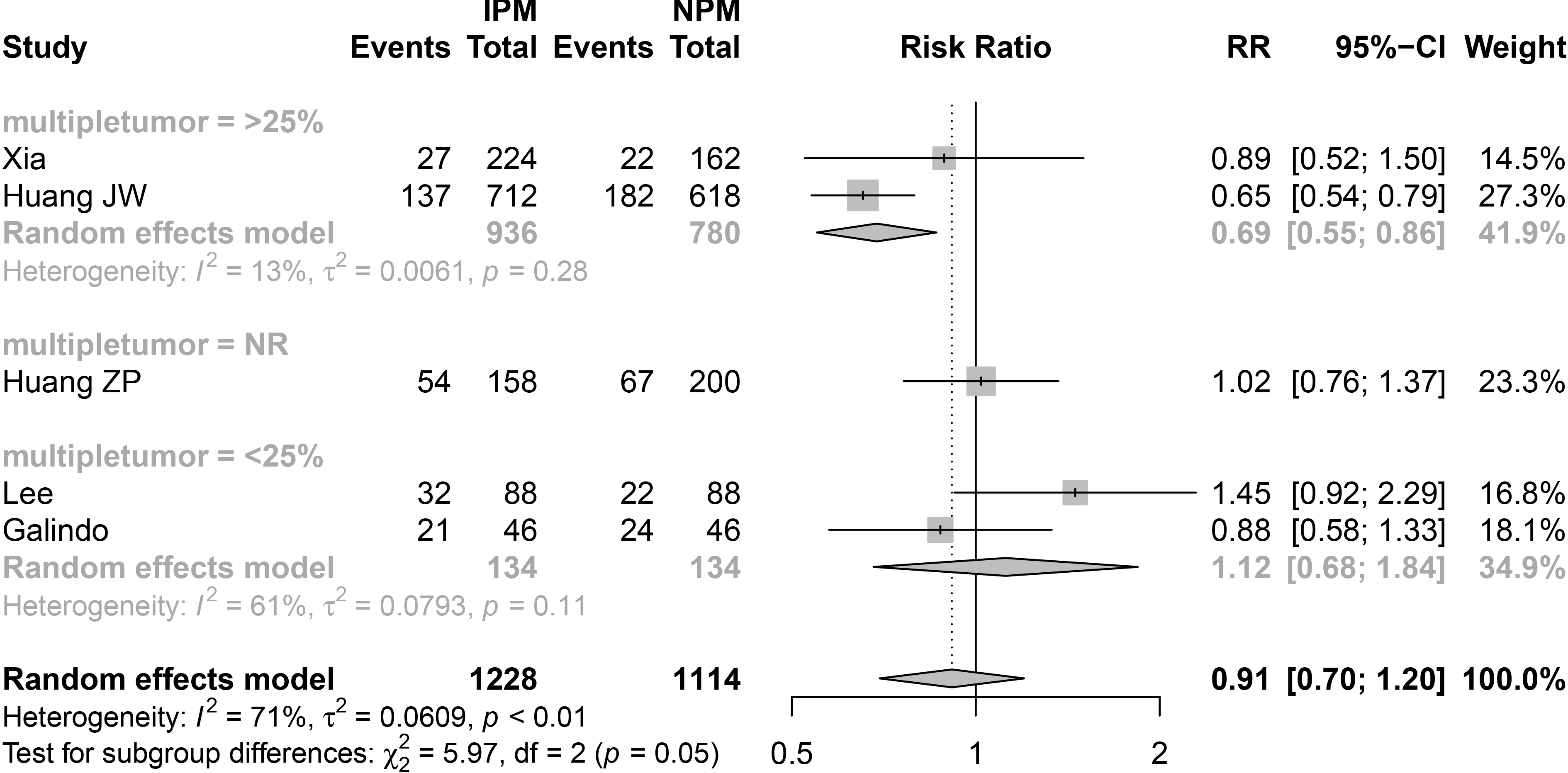


E


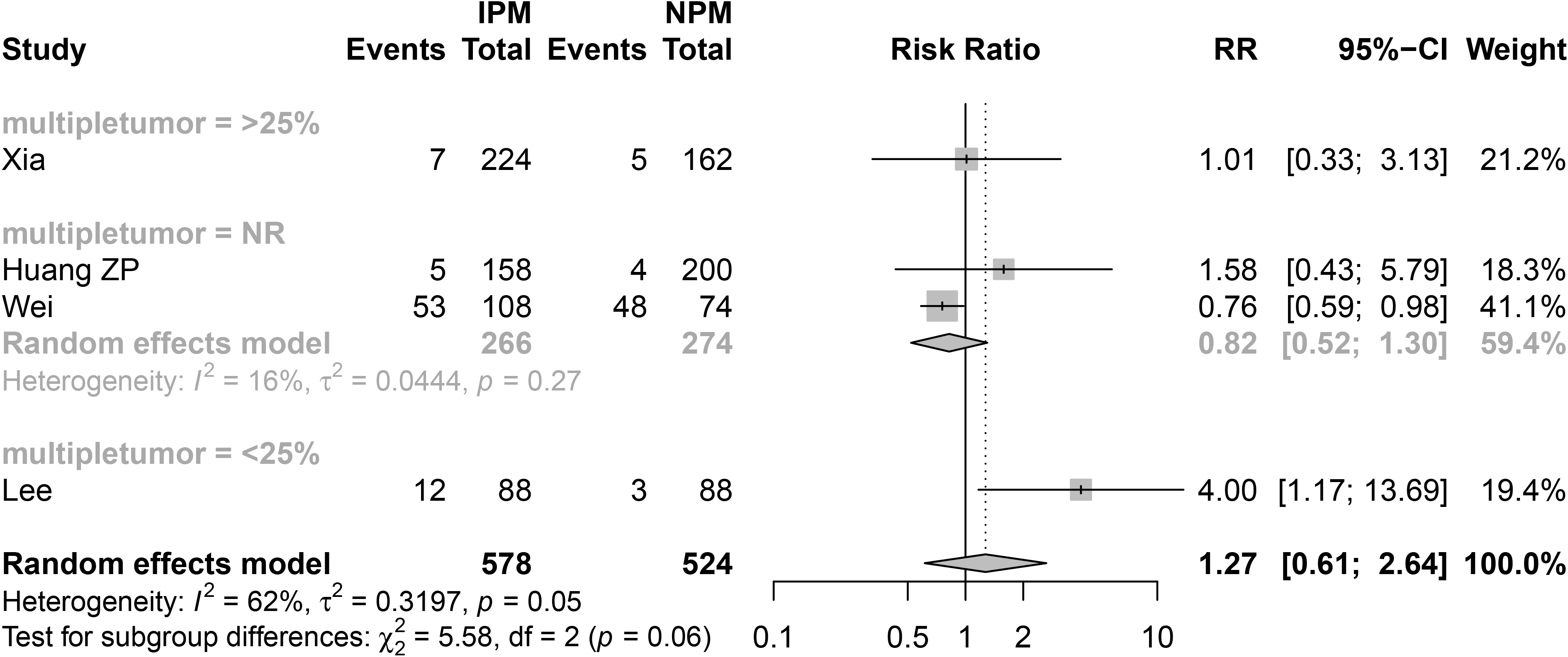


F


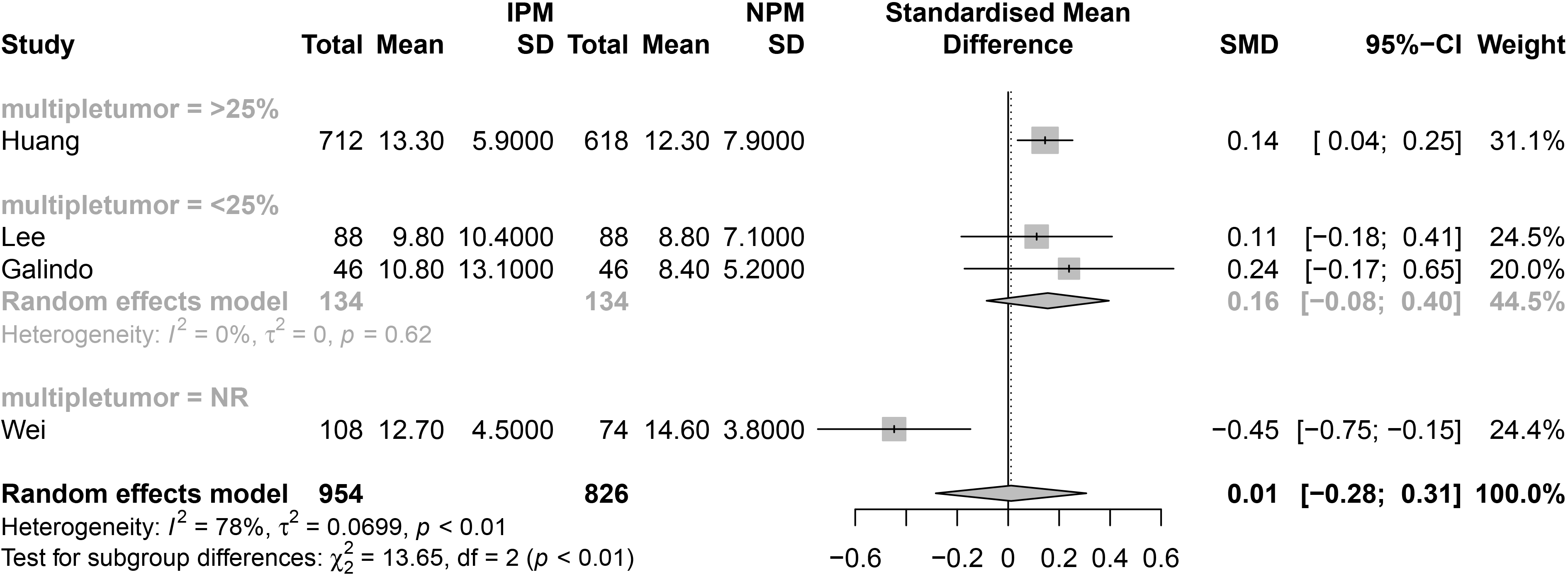

Supplement: Supplementary file 1 — Additional file 1: Supplementary Material 1. Search strategy. Supplementary Material 2. Risk assessment of RCT. Supplementary Material 3. NOS score of non-RCT studies. Supplementary Material 4. Subgroup analysis for overall survival and disease-free survival. Supplementary Material 5. Sensitivity analyses for overall survival and disease-free survival. Supplementary Material 6. Funnel plot for overall survival and disease-free survival. A, overall survival; B, disease-free survival. Supplementary Material 7. The effect of publication bias evaluated by using the trim and fill method for overall survival and disease-free survival. Supplementary Material 8. Forest plot after trimming and filling for overall survival and disease-free survival. Supplementary Material 9. Forest plot of sensitivity analysis for blood loss. Supplementary Material 10. Forest plot for blood loss after omitting the study by Fumularo et al. Supplementary Material 11. Forest plot of sensitivity analysis for blood loss after omitting the study by Fumularo et al. Supplementary Material 12. Forest plot of subgroup analysis based on the proportion of patients with Child A, the cut value was 90%. A, operation time; B, blood loss; C, blood transfusion; D, total complication; E, pleural effusion; F, hospital stay. Supplementary Material 13. Forest plot of subgroup analysis based on the proportion of patients with liver cirrhosis, the cut value was 70% for operation time and blood transfusion, while 60% for the rest. A, operation time; B, blood loss; C, blood transfusion; D, total complication; E, pleural effusion; F, ascites; G, hospital stay. Supplementary Material 14. Forest plot of subgroup analysis based on the proportion of patients received major liver resection, the cut value were 40% and 60% for blood loss, while 40% for the reset. A, operation time; B, blood loss; C, blood transfusion; D, total complication; E, hospital stay. Supplementary Material 15. Forest plot of subgroup analysis based on [file 12957_2023_3244_MOESM1_ESM.docx]
